# Efficacy and safety of laser acupuncture on osteoarthritis: a systematic review and meta-analysis

**DOI:** 10.3389/fnagi.2024.1462411

**Published:** 2025-01-08

**Authors:** Xiangdong Wen, Guojiang Zhang, Jinquan Cui, Yuzhe Tang, Qi Meng, Yang Su, Senbo An, Shui Sun

**Affiliations:** ^1^Department of Joint Surgery, Shandong Provincial Hospital, Shandong University, Jinan, Shandong, China; ^2^Department of Joint Surgery, Shandong Provincial Hospital Affiliated to Shandong First Medical University, Jinan, Shandong, China

**Keywords:** laser acupuncture, osteoarthritis, pain, VAS (visual analog scale), meta-analysis

## Abstract

**Objectives:**

To perform a meta-analysis of previous studies investigating the effects of laser acupuncture on osteoarthritis.

**Study design:**

Systematic review and meta-analysis.

**Methods:**

Randomized controlled trials (RCTS) on laser acupuncture for osteoarthritis were searched in the databases of PubMed, Embase, Cochrane Library, and Web of Science with a search deadline of 24 December 2023. After identifying 11 studies, we used Stata 15.0 to analyze the data.

**Results:**

In the 11 studies identified, 931 patients were analyzed. Results showed that laser acupuncture significantly improved patients’ pain and function compared to the placebo laser group. There were significant differences in VAS pain scores[SMD = −0.924, 95% CI (−1.200, −0.649), *p* = 0.000], WOMAC pain scores[SMD = −0.425, 95% CI (−0.652, −0.199), *p* = 0.000], WOMAC function scores[SMD = −0.307, 95% CI (−0.548, −0.065), *p* = 0.013], WOMAC stiffness scores[SMD = −0.235, 95% CI (−0.388, −0.083), *p* = 0.002] between the laser acupuncture group and the placebo laser group. The therapeutic effect of laser acupuncture disappeared at 8 weeks. In subgroup analysis, patients who received laser acupuncture with specific parameters had better VAS scores and WOMAC scores than patients in other subgroups.

**Conclusion:**

The application of laser acupuncture can improve knee pain and function in patients with osteoarthritis in the short term. It is recommended to use a laser with a power greater than 100 mW and a wavelength greater than 1,000 nm. CO2 lasers and solid-state lasers were shown to be more effective in the results than other types of lasers.

## 1 Introduction

Osteoarthritis (OA) is a chronic degenerative disease characterized by degenerative injury of articular cartilage and osteophytic hyperplasia of the articular edge, which is the main cause of disability in the elderly (Bijlsma et al., 2011; Felson et al., 2000). It often causes joint pain, swelling, stiffness, and limited mobility, and in severe cases can lead to joint deformity (Mahmoudian et al., 2021). There were 527.8 million prevalent cases and 41.5 million incident cases worldwide in 2019 (Cao et al., 2024). With the trend of an aging population, an increasing proportion of obese individuals, and a rising incidence of joint injuries, the disease and economic burden caused by osteoarthritis have significantly increased (Hunter et al., 2014; Wong et al., 2023). Because damaged articular cartilage cannot be restored, the treatment of osteoarthritis aims to relieve pain, delay disease progression, improve joint function, and improve quality of life. Treatment strategies include physical therapy, drug therapy, exercise therapy, and surgery (Glyn-Jones et al., 2015; Kong et al., 2022; McAlindon et al., 2014).

Laser acupuncture is a new kind of acupuncture method that uses a fine beam of laser to irradiate acupoints to treat diseases. Compared with traditional acupuncture, laser acupuncture has no pain caused by acupuncture (Baxter et al., 2008), and there is no possibility of needle breaking and acupuncture site infection (Li, 2015). It can dredge the meridians, promote blood circulation, and relieve pain. Low-level laser action on mitochondria increases adenosine triphosphate (ATP) production, reactive oxygen species (ROS) regulation, and transcription factor induction. Transcription factors then promote relevant protein synthesis, triggering further downstream effects such as increasing cell proliferation and migration, and regulating levels of cytokines, growth factors, and inflammatory mediators (Chung et al., 2012). Some studies have shown that laser acupuncture has the potential to treat inflammatory diseases, temporomandibular disorders, schizophrenia, and other diseases (Attia et al., 2016; Mota et al., 2024; Shen et al., 2014).

The purpose of this review was to conduct a meta-analysis of previous RCTS of laser acupuncture to evaluate the efficacy of laser acupuncture in the treatment of osteoarthritis and to explore the influence of laser parameters on the efficacy of treatment.

## 2 Methods

The systematic review described herein was accepted by the online PROSPERO International Prospective Register of Systematic Reviews of the National Institute for Health Research (Page et al., 2021). Prospero Registration Number: CRD42024555934.

### 2.1 Inclusion and exclusion criteria

The included population met the diagnostic criteria for osteoarthritis (Altman et al., 1986; McAlindon and Dieppe, 1989), regardless of age and sex. Laser acupuncture was used in the experimental group and the control group was either placebo or sham laser. We identified studies that used at least one of the selected outcomes—knee pain scores [the Western Ontario and McMaster Universities Arthritis Index (WOMAC) pain scores or the visual analog scale [VAS] pain scores] or any knee functional scores—at a variety of time points after laser acupuncture. Only randomized controlled studies were included, while duplicate publications, conference abstracts, replies and comments, meta-analyses and reviews, case reports, and full text were not available, as well as animal experiments, were excluded from the analysis.

### 2.2 Literature retrieval

Randomized controlled trials on laser acupuncture for arthritis were searched in PubMed, Embase, Cochrane Library, and Web of Science. The search deadline was 24 December 2023, using the mesh word combined with a free word: laser acupuncture and osteoarthritis. Detailed search strategies are provided in Supplementary material 1.

### 2.3 Data extract

Two reviewers rigorously screened the literature based on predetermined inclusion and exclusion criteria. In case of any disagreement, they resolved it through discussion or sought the opinion of a third party to negotiate and reach a consensus. Information extracted from the included studies included the following key details: authors, year, country, sample size (experimental groups and the control groups), age, gender, intervention, and outcome.

### 2.4 Included studies’ risk of bias

Two reviewers independently assessed the risk of bias as low, unclear, or high using the Cochrane Collaboration’s tools (Higgins et al., 2011). Disagreements were resolved by consensus and discussion with a third reviewer. The assessment included seven areas: generation of randomized sequences (selective bias), allocation concealment (selective bias), blinding of implementers and participants (implementation bias), blinding of outcome assessors (observational bias), completeness of outcome data (follow-up bias), selective reporting of study results (reporting bias), and other potential sources of bias. Each included study was assessed individually against these criteria. If a study fully met all criteria, it was at “low risk” of bias, indicating a high-quality study and low overall risk of bias. If a study partially met the criteria, its quality was categorized as ‘unclear risk’, indicating a moderate likelihood of bias. If a study did not meet the criteria at all, it was categorized as “high risk,” indicating a high risk of bias and low quality of the study.

### 2.5 Data analysis

The collected data were statistically analyzed using Stata 15.0 software (Stata Corp., College Station, TX, United States). Heterogeneity between included studies was assessed using I2 values or Q-statistics. I2 values of 0, 25, 50, and 75% indicated no heterogeneity, low heterogeneity, moderate heterogeneity, and high heterogeneity, respectively. If the I2 value was equal to or greater than 50%, a sensitivity analysis was performed to explore potential sources of heterogeneity. If heterogeneity was less than 50 percent, analyses were conducted using a fixed-effects model. Standardized mean difference (SMD) and 95% confidence interval (CI) were used for continuous variables and odds ratio (OR) and 95% confidence interval (CI) for dichotomous variables. In addition, Begg’s test and Egger’s test were used to assess publication bias. The significance level of the meta-analysis was *α* = 0.05, and *p* < 0.05 indicated that the comparative difference between the results of each study was statistically significant.

## 3 Results

### 3.1 Study selection

**Figure 1:**
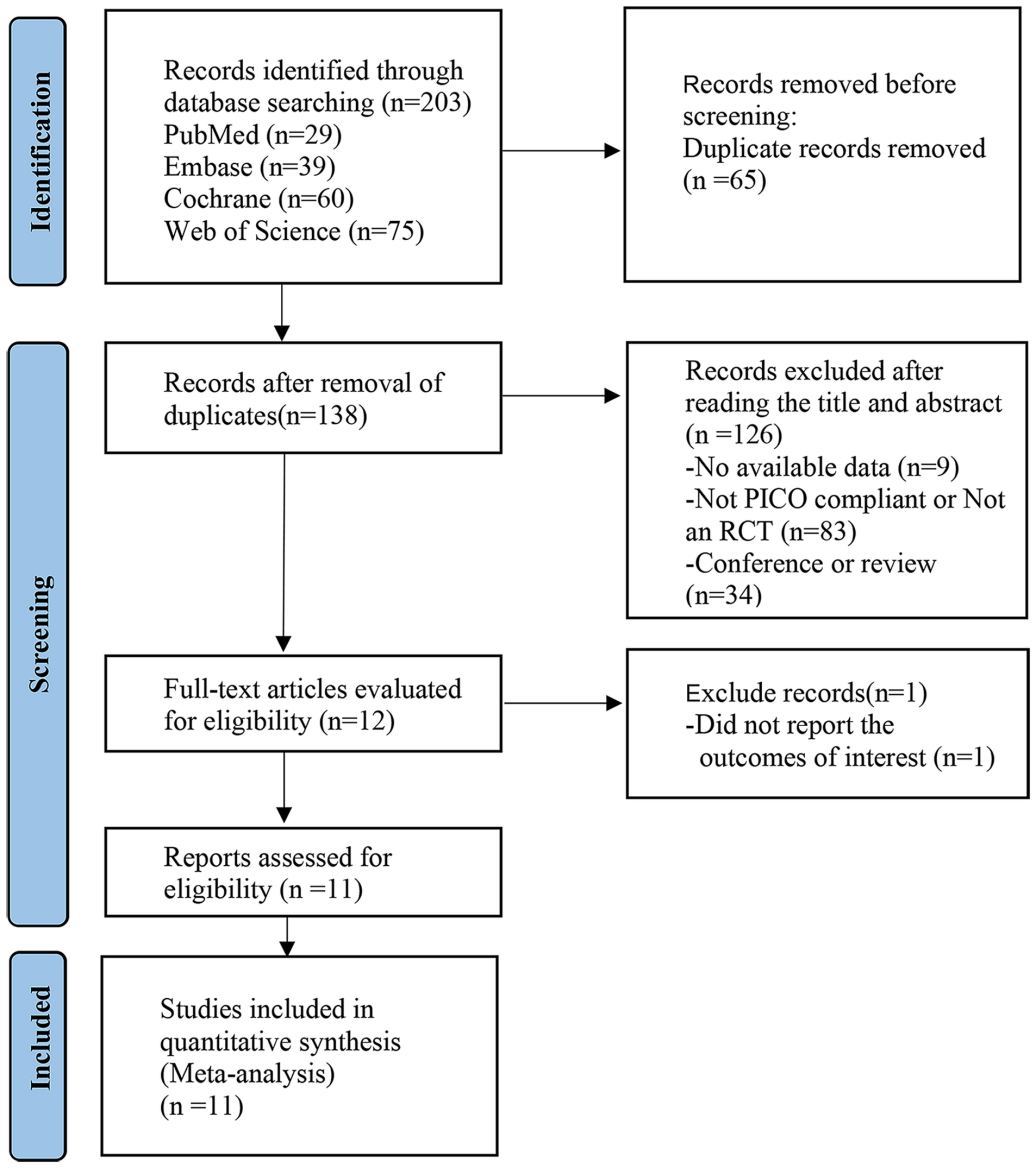
PRISMA flow diagram of the study process. PRISMA, Preferred Reporting Items for Systematic Review and Meta-analysis.

Figure 1 shows our literature search process, which initially retrieved 203 articles, removed 65 duplicates, removed 126 articles by reading titles and abstracts, removed 1 article by reading the full text, and finally included 11 randomized controlled trials (Al Rashoud et al., 2014; Helianthi et al., 2016; Hinman et al., 2014; Kheshie et al., 2014; Liao et al., 2020; Mohammed et al., 2018; Ren et al., 2010; Shen et al., 2009; Wu et al., 2011; Yurtkuran et al., 2007; Zhao et al., 2021) for analysis.

### 3.2 Basic characteristics and risk of bias of the included studies

**Figure 2:**
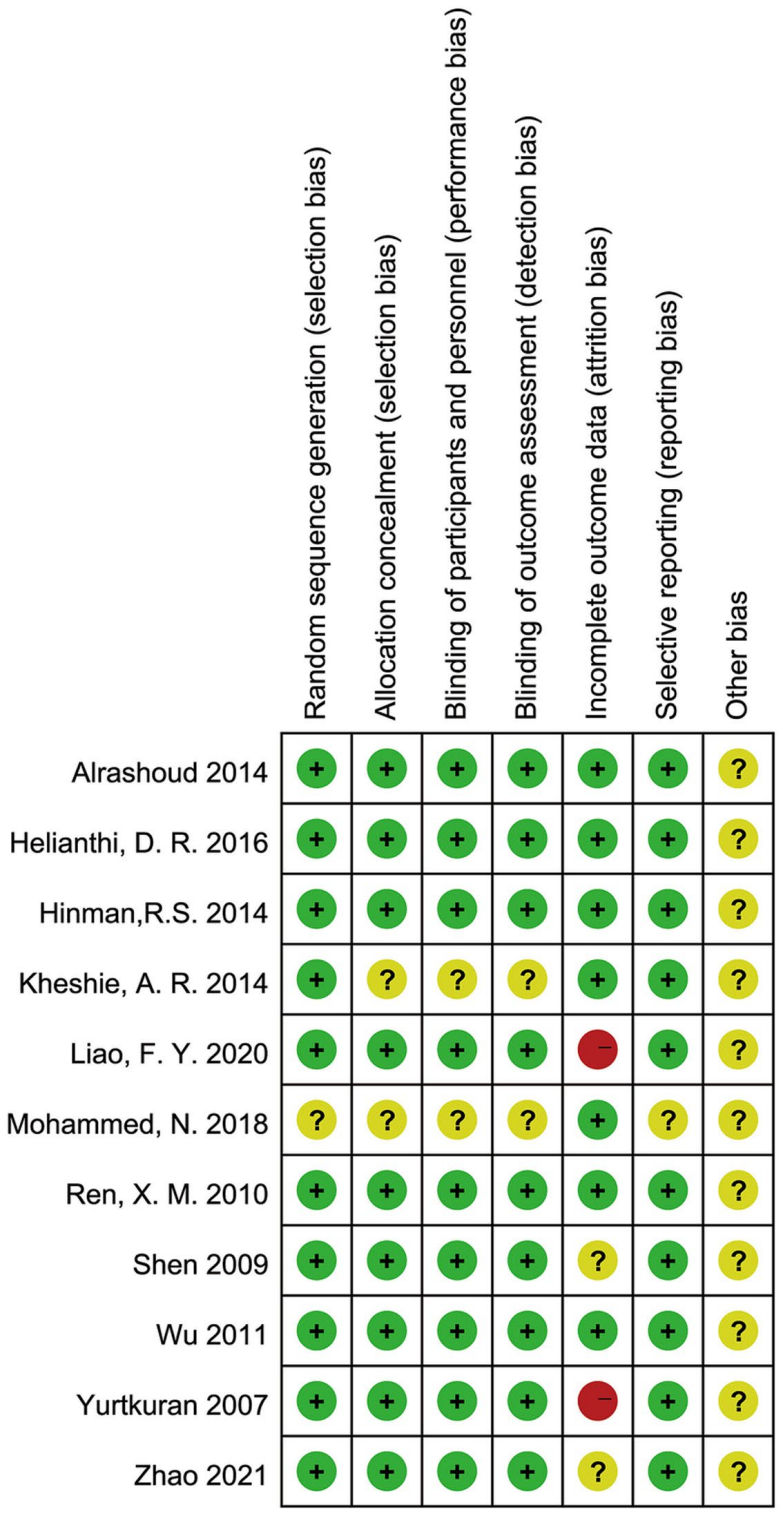
The risk of bias in studies.

Eleven randomized controlled studies involving 931 individuals were finally included, 486 in the Laser therapy group and 445 in the sham laser group. In total, five studies were conducted in China, others were performed in the UK, Indonesia, Egypt, Turkey, Saudi Arabia, and Australia. Average age ranged from 50 to 70 years old and follow-up period ranged from 1 week to 1 year. Basic characteristics are shown in Table 1. The 10 included studies clearly accounted for the method of randomization used. The random sequence generation of one article was not adequately illustrated. The details of the risk of bias assessment can be obtained in Figure 2.

**Table 1:** Basic characteristics of included studies.

Study	Year	Country	Sample size		Gender (M/F)	Mean age		Intervention		Outcome
			EG	CG		EG	CG	EG	CG	
Alrashoud	2014	UK	26	23	18/31	52	56	LA:1.Output power: 30 mW 2.Wavelength: 830 nm 3.Acupoints: EX-LE5, SP9, SP10, ST35, ST36 4.Duration: 40s 5. Frequency:/6.Total time:/7.Device: gallium aluminum arsenide laser device with a single 30-mW diode probe	Placebo	VAS
Helianthi, D. R.	2016	Indonesia	30	29	17/42	69	68	LA:1.Output power: 50 mW 2.Wavelength 0.785 μm 3.Acupoints: ST35, ST36, SP9, GB34, EX-LE4 4.Duration: 80s 5.Frequency: 2 times per week 6.Total time:5 weeks 7.Device: single-probe gallium aluminum arsenide laser device	Placebo	VAS
Liao, F. Y.	2020	China	15	15	3/27	70.53	69.73	LA:1.Output power: 50 mW and 30 mW 2.Wavelength: 0.78 μm and 0.83 μm 3.Acupoints: SP9, SP10, EX-LE2 4.Duration: 15 min 5.Frequency: 3 times per week 6.Total time:4 weeks 7.Device: multiband laser therapeutic apparatuses (TI-816-2, Transverse Inc., Taiwan)	Placebo	VAS
Mohammed, N.	2018	Egypt	20	20	6/34	55.25	50.11	LA:1.Output power:90 mW 2.Wavelength: 0.808 μm 3.Acupoints: SP9, SP10, ST35, ST36, GB34, Ashi points 4.Duration: 1 min (Ashi points 4 min) 5.Frequency: 3 times per week 6.Total time:4 weeks 7.Device: Semiconductor (diode laser) Galuim Aluminum Arsenide (GA-AL-AS)	Sham	VAS
Ren, X. M.	2010	China	22	19	9/32	61.8	59.9	LA:1.Output power:36 mW and 200 mW 2.Wavelength: 0.65 ~ 0.66 μm and 10.6 μm 3.Acupoints: ST35,Ashi points 4.Duration: 20 min 5.Frequency: 3 times per week 6.Total time:4 weeks 7.Device: Semiconductor laser generator and CO2 laser generator	Placebo	WOMAC
Shen	2009	China	20	20	4/36	60.10	56.40	LA:1.Output power: 36 mW and 200 mW 2. Wavelength: 0.65 ~ 0.66 μm and 10.6 μm 3.Acupoints: ST35 4. Duration: 20 min 5.Frequency: 3 times per week 6.Total time:4 weeks 7.Device: 650 nm semiconductor laser	Placebo	WOMAC
Wu	2011	China	15	16	8/23	63	63.5	LA:1.Output power: 60-65 mW 2.Wavelength: 10.6 μm 3.Acupoints: ST35 4. Duration: 20 min 5.Frequency: 3 times per week 6.Total time: 4 weeks 7.Device: CO2 laser generator	Sham	SF-36 scale
Yurtkuran	2007	Turkey	28	27	2/53	51.83	53.478	LA:1.Output power:4 mW 2.Wavelength: 0.904 μm 3.Acupoints: SP9 4. Duration: 20 min 5. Frequency: 5 times per week 6. Total time: 2 weeks 7.Device:(Infrared 27 GaAs diode laser instrument)	Placebo	VAS、WOMAC
Zhao	2021	China	201	191	98/294	63.5	63.1	LA:1.Output power:160–180 mW 2.Wavelength: 10.6 μm 3.Acupoints: ST35 4. Duration: 20 min 5.Frequency: 3 times per week 6. Total time:4 weeks 7.Device: CO2 laser generator	Sham	VAS、WOMAC、SF-36
Hinman, R.S.	2014	Australia	71	70	74/67	63.4	63.8	LA:1.Output power:10 mW 2.Wavelength:/3. Acupoints:SP9, 10 ST34, 35, 36 LR7, 8, 9 KI10 BL39, 40, 57 GB34, 35, 36 4.Duration: 20 min 5.Frequency: 1 time or 2 times per week 6.Total times: 8–12 times 7.Device: semiconductor laser	Sham	WOMAC
Kheshie, A. R.	2014	Saudi Arabia	LLLT 18	15	33/0	56.56	55.6	LA:1.Output power 800 mW 2.Wavelength: 830 nm 3.Acupoints:/4.Duration:32 min and 33 s 5.Frequency:2 times per week 6.Total time: 6 weeks 7.Device: semiconductor laser	Placebo	VAS、WOMAC
			HILT 20	15	35/0	52.1	55.6	LA:1.Output power:10.5w 2.Wavelength: 1064 nm 3.Acupoints/4.Duration:15 min 5.Frequency:2 times per week 6.Total time:6 weeks 7.Device: Solid-state laser	Placebo	VAS、WOMAC

EG, experimental group; CG, control group; M/F, gender ratio; LA, laser acupuncture; HILT, high-intensity laser therapy; LLLT, low-level laser therapy.

### 3.3 Result of meta-analysis

#### 3.3.1 VAS

**Figure 3:**
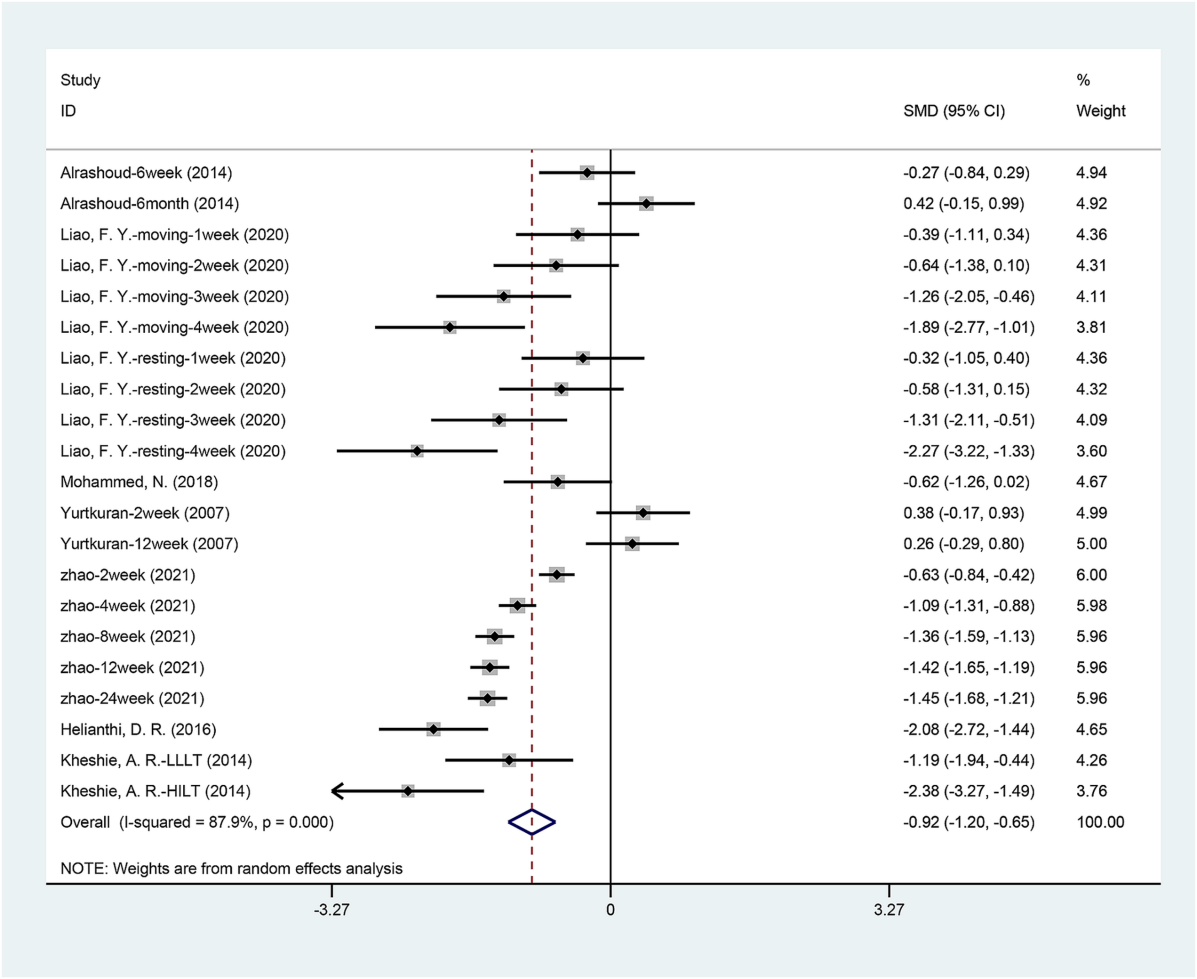
Forest plot of effects of laser acupuncture on knee pain (VAS pain scores).

Seven articles (Al Rashoud et al., 2014; Hinman et al., 2014; Kheshie et al., 2014; Liao et al., 2020; Mohammed et al., 2018; Yurtkuran et al., 2007; Zhao et al., 2021) mentioned the change in VAS pain scores. The combined data showed that the VAS of patients receiving laser acupuncture was significantly lower than the patients receiving sham or placebo laser therapy [SMD = −0.924, 95% CI (−1.200, −0.649), *p* = 0.000] (Figure 3). Significant heterogeneity was found among the enrolled studies [I2 = 87.9%, *p* = 0.000]. Due to the significant heterogeneity, we performed a sensitivity analysis using a one-by-one approach, and the results of the analysis showed a low sensitivity and stable results for this outcome (Supplementary Figure S1).

#### 3.3.2 WOMAC pain scores

**Figure 4:**
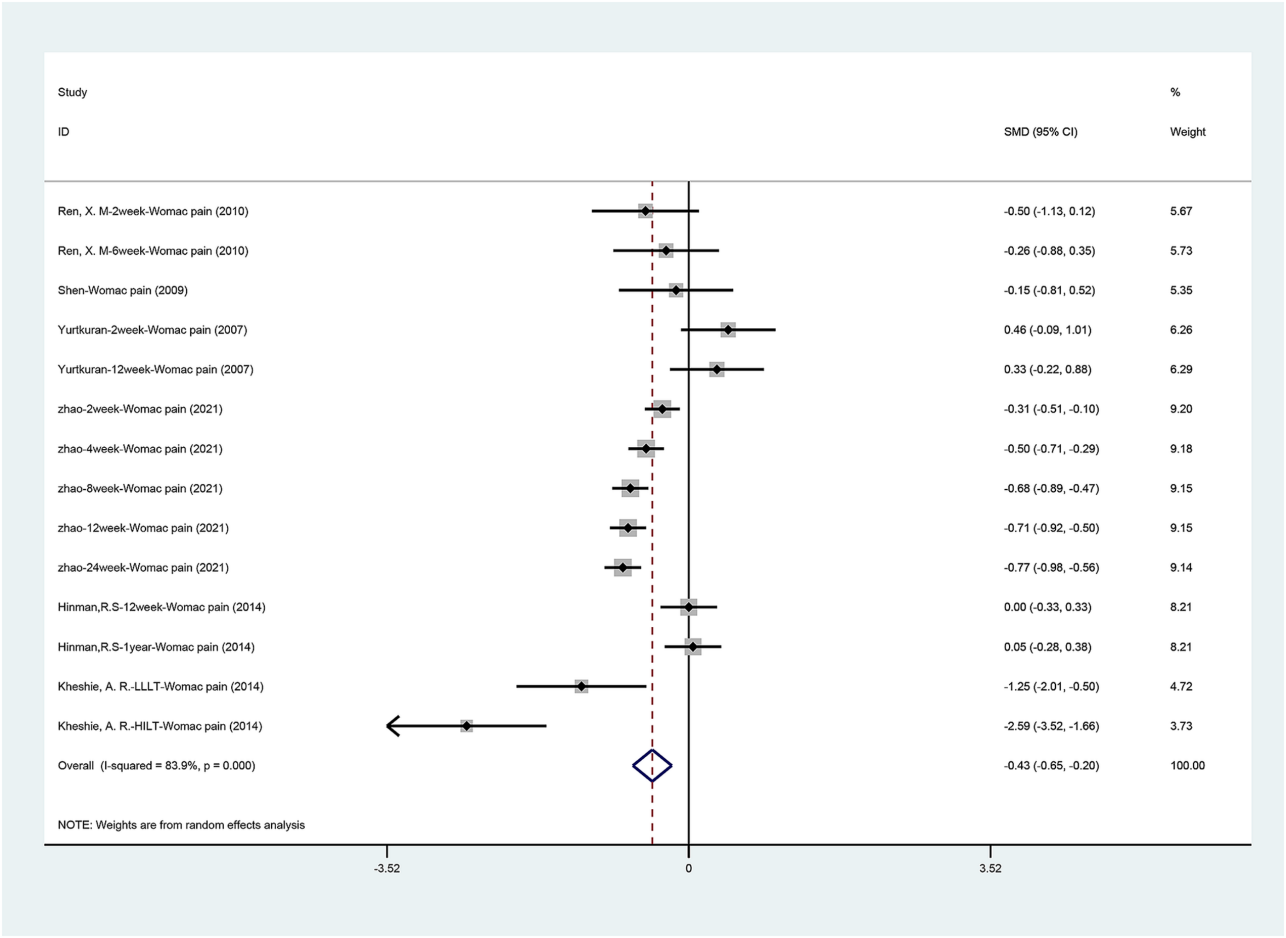
Forest plot of effects of laser acupuncture on knee pain (WOMAC pain scores).

Figure 4 summarizes the WOMAC pain scores comparing laser therapy with sham laser therapy. Six articles (Hinman et al., 2014; Kheshie et al., 2014; Ren et al., 2010; Shen et al., 2009; Yurtkuran et al., 2007; Zhao et al., 2021) mentioned the change in WOMAC pain scores. The results are similar to VAS pain scores, laser acupuncture can significantly reduce pain in patients with osteoarthritis [SMD = −0.425, 95% CI (−0.652, −0.199), *p* = 0.000]. Significant heterogeneity was found among the enrolled studies (I2 = 83.9%, *p* = 0.000). Due to the significant heterogeneity, we performed a sensitivity analysis using a one-by-one approach, and the results of the analysis showed a low sensitivity and stable results for this outcome (Supplementary Figure S2).

#### 3.3.3 WOMAC function scores

**Figure 5:**
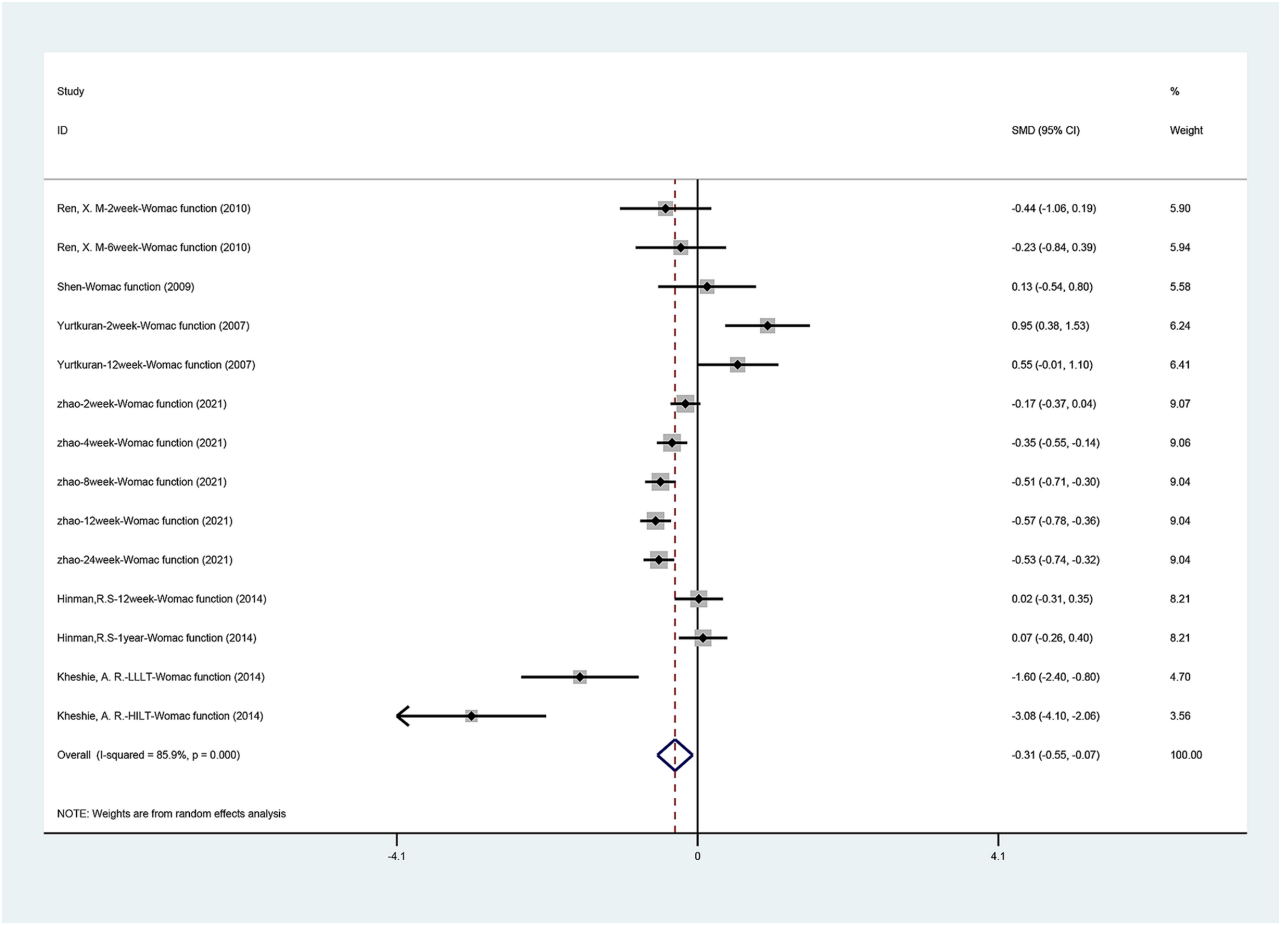
Forest plot of effects of laser acupuncture on knee function (WOMAC function scores).

The WOMAC function scores comparing laser therapy with sham laser therapy are available in Figure 5. A total of six studies (Hinman et al., 2014; Kheshie et al., 2014; Ren et al., 2010; Shen et al., 2009; Yurtkuran et al., 2007; Zhao et al., 2021) described the function recovery evaluated by the WOMAC function scores. Statistical heterogeneity was observed in the present meta-analysis (I2 = 85.9%, *p* = 0.000); therefore, a random-effects model was applied. The pooled results revealed laser therapy provided a better function recovery than sham laser [SMD = −0.307, 95% CI (−0.548, −0.065), *p* = 0.013]. Due to the significant heterogeneity, we performed a sensitivity analysis using a one-by-one approach, and the results of the analysis showed a low sensitivity and stable results for this outcome (Supplementary Figure S3).

#### 3.3.4 WOMAC stiffness scores

**Figure 6:**
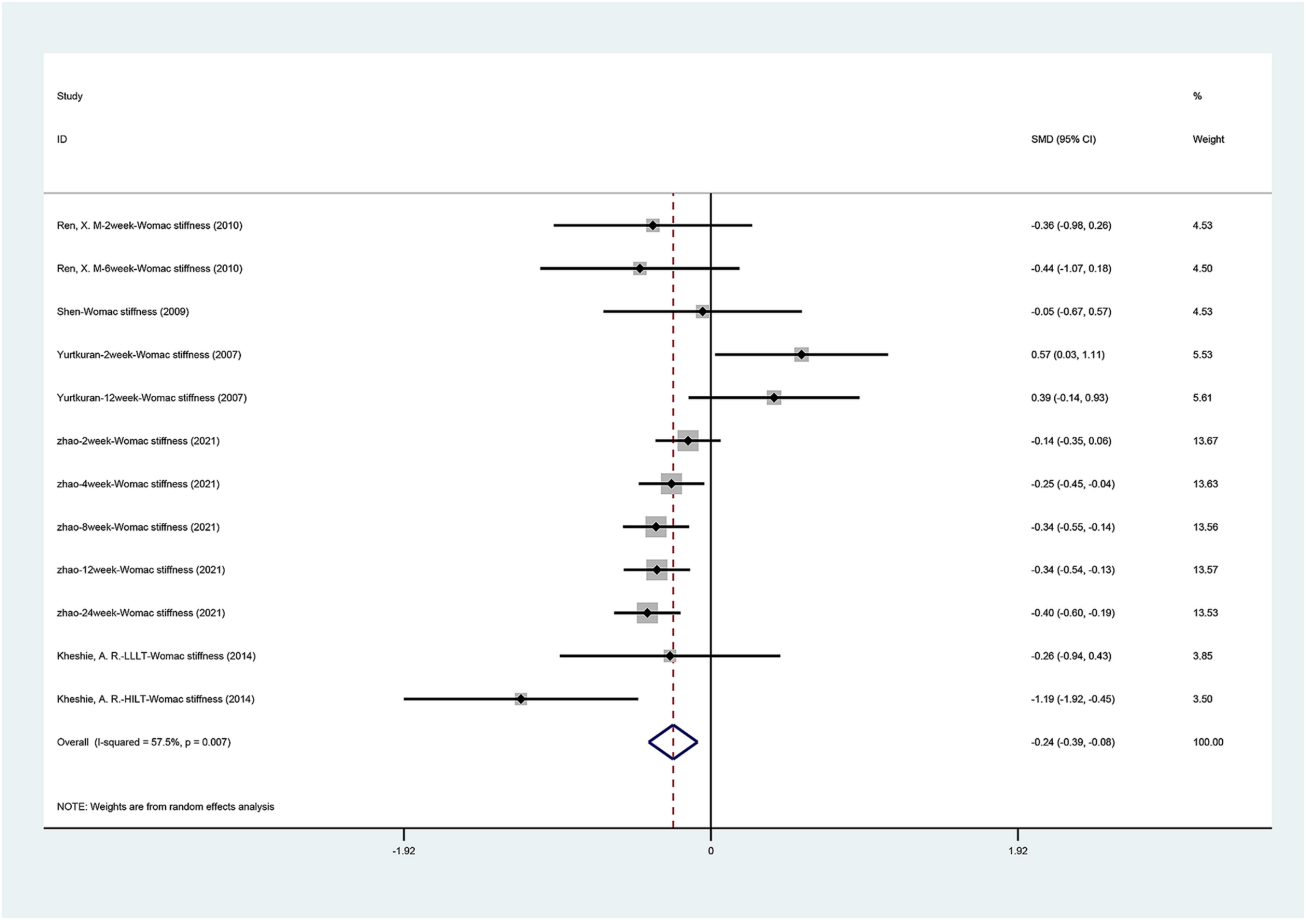
Forest plot of effects of laser acupuncture on knee function (WOMAC stiffness scores).

Figure 6 presents the WOMAC stiffness scores comparing laser acupuncture with sham laser acupuncture. A total of five studies (Kheshie et al., 2014; Ren et al., 2010; Shen et al., 2009; Yurtkuran et al., 2007; Zhao et al., 2021) showed the WOMAC stiffness scores. There was a statistically significant difference between laser acupuncture and sham laser acupuncture for the WOMAC stiffness scores [SMD = −0.235, 95% CI (−0.388, −0.083), *p* = 0.002]. Laser acupuncture showed effectiveness in improving knee stiffness. Significant heterogeneity was found among the enrolled studies (I 2 = 57.5%, *p* = 0.007). Due to the significant heterogeneity, we performed a sensitivity analysis using a one-by-one approach, and the results of the analysis (Supplementary Figure S4) showed a low sensitivity and stable results for this outcome.

#### 3.3.5 SF-36 scale

**Figure 7:**
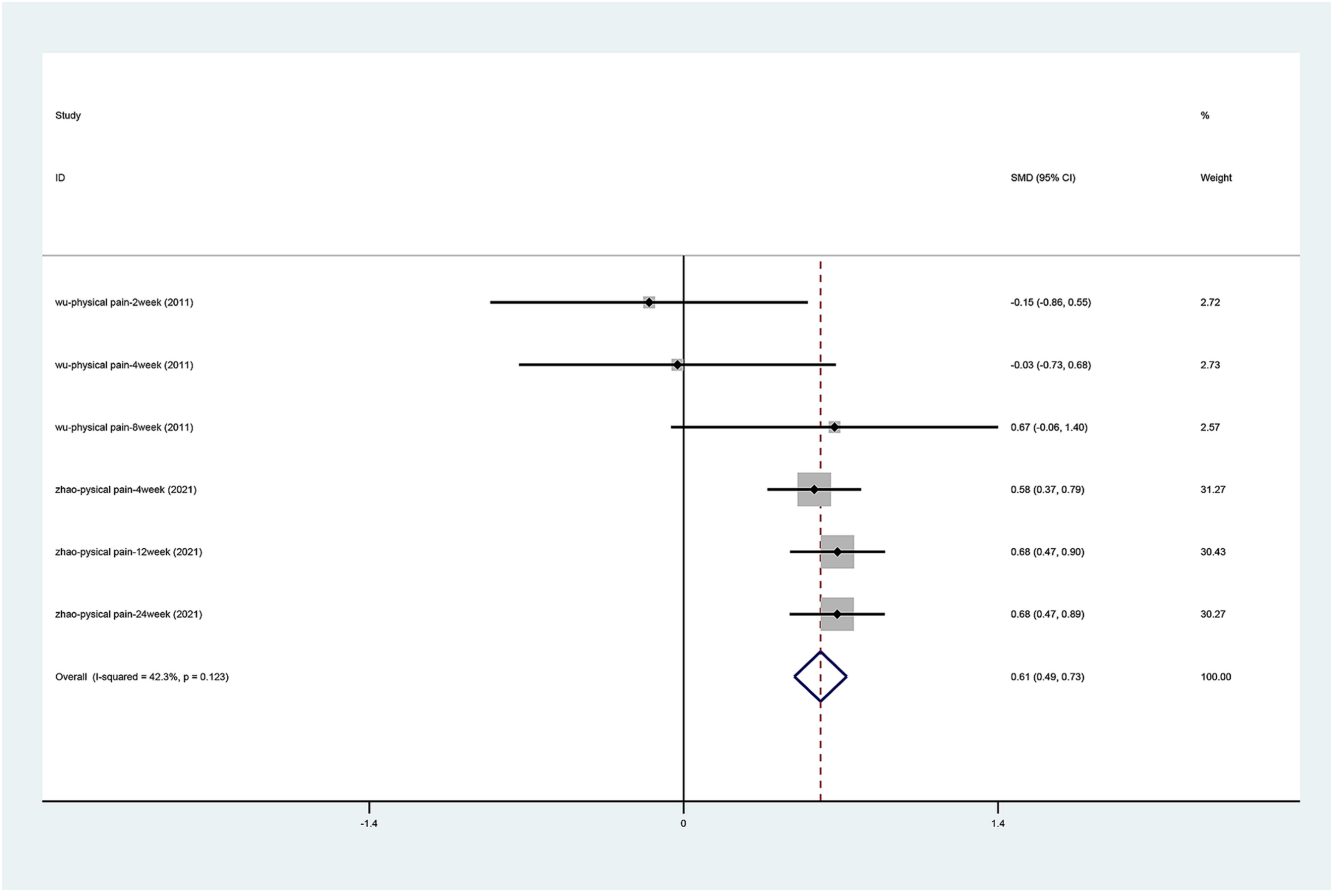
Forest plots of effects of laser acupuncture on knee SF-36 scale (physical pain).

**Figure 8:**
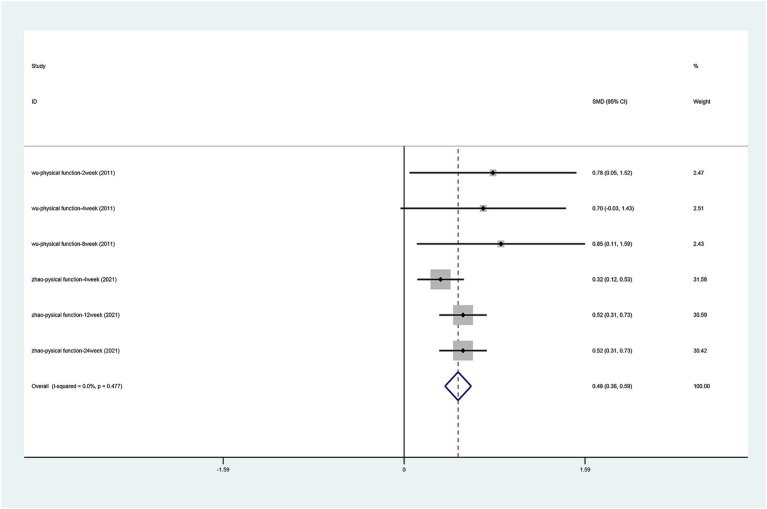
Forest plots of effects of laser acupuncture on knee SF-36 scale (physical function).

There were only two articles (Wu et al., 2011; Zhao et al., 2021) that mentioned the change in physical pain and physical function on the SF-36 scale. After the heterogeneity test (physical pain I2 = 42.3%, *p* = 0.123, physical function I2 = 0.0%, *p* = 0.477), the fixed effects model was used. The results of the analysis (Figures 7, 8) suggest that laser acupuncture was able to improve physical pain [SMD = 0.611, 95% CI (0.498, 0.724), *p* = 0.000] and physical function [SMD = 0.476, 95% CI (0.365, 0.588), *p* = 0.000].

#### 3.3.6 Time-dependent effects of laser acupuncture

**Figure 9:**
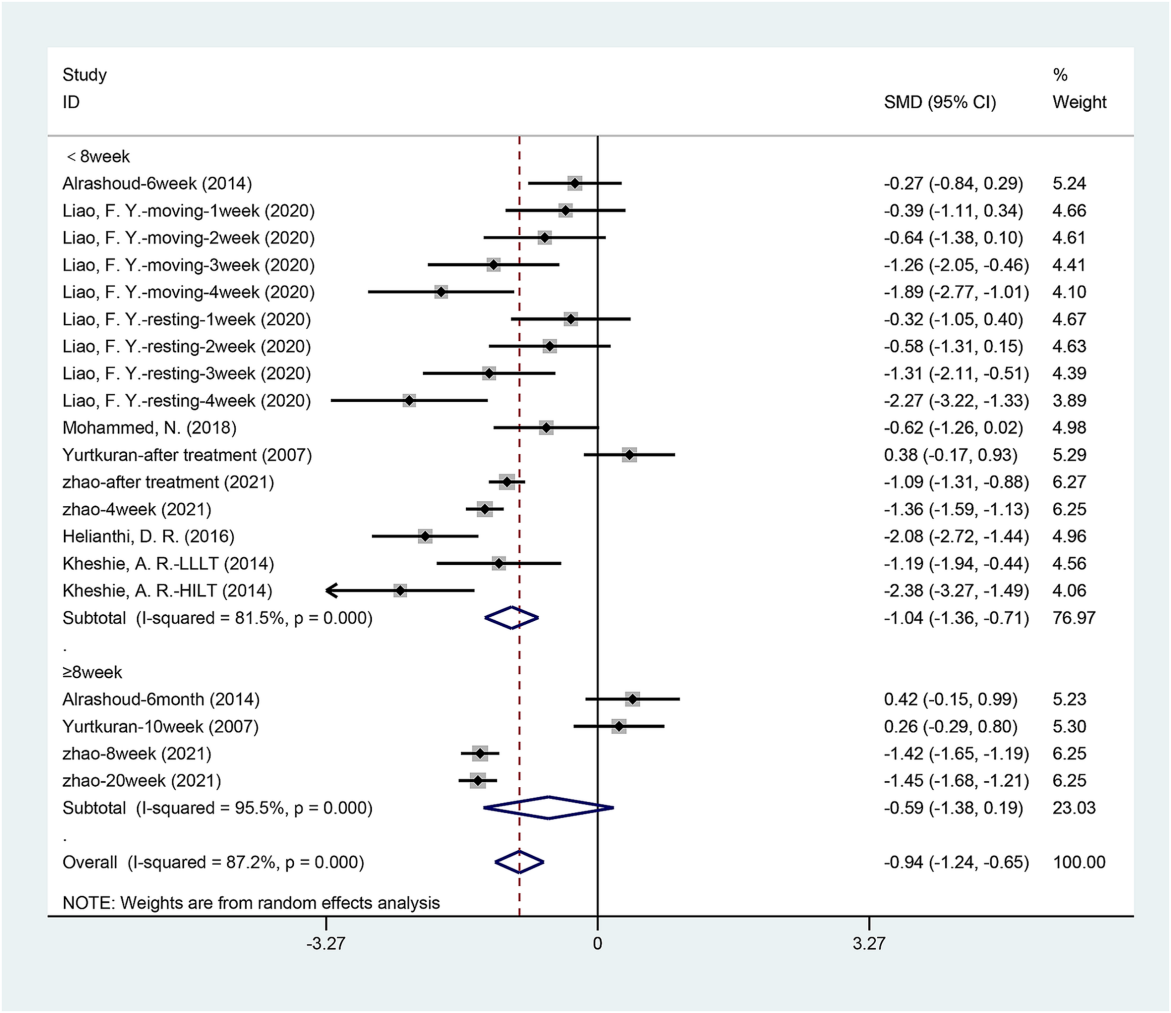
Subgroup analysis of VAS pain scores at different follow-up times.

We tried to explore how the therapeutic effects of laser acupuncture have changed over time. We performed a subgroup analysis based on the timing of the outcome assessment. Figure 9 shows the VAS pain scores of patients within 8 weeks and 8 weeks later after the end of treatment. As shown in the figure, there was a significant difference between laser treatment and sham laser treatment when followed up within 8 weeks (< 8 weeks) after the end of treatment [SMD = −1.038, 95% CI (−1.362, −0.715), p = 0.000], but the difference between the two groups disappeared when followed up 8 weeks later (≥ 8 weeks) [SMD = −0.593, 95% CI (−1.379, 0.193), *p* = 0.139].

Consistent with the VAS results, WOMAC pain scores and WOMAC function scores showed the same results (Table 2). There was a significant difference between laser treatment and sham laser treatment when followed up within 8 weeks (< 8 weeks) after the end of treatment. There was no statistically significant difference in WOMAC pain scores [SMD = −0.325, 95% CI (−0.770, 0.120), *p* = 0.152] and WOMAC function scores [SMD = -0.185, 95% CI (−0.582, 0.213), *p* = 0.363] between the two groups at follow-up 8 weeks after the end of treatment. There were no statistically significant differences in WOMAC stiffness scores between the two subgroups (< 8 weeks [SMD = −0.278, 95% CI (−0.569, 0.013), *p* = 0.061], ≥ 8 weeks [SMD = -0.224, 95% CI (−0.525, 0.077), *p* = 0.145]). The improvement effect of laser acupuncture on VAS pain scores, WOMAC pain scores, and WOMAC function scores of patients disappeared at 8 weeks.

**Table 2:** Time-dependent effects of laser acupuncture in WOMAC pain scores, WOMAC function scores, WOMAC stiffness scores.

Outcome	Follow-up	No. of trials	Standard mean difference (95% CI)	*p*-value	I2(%)	I2 *P*-value
WOMAC pain scores	<8 weeks	5	−0.569(−0.978, −0.159)	0.006	87.3%	0.000
	≥8 weeks	3	−0.325(−0.770, 0.120)	0.152	89.6%	0.000
WOMAC function scores	<8 weeks	5	−0.531(−1.007, −0.056)	0.028	90.6%	0.000
	≥8 weeks	3	−0.185(−0.582, 0.213)	0.363	87.0%	0.000
WOMAC stiffness scores	<8 weeks	4	−0.278(−0.569, 0.013)	0.061	67.8%	0.008
	≥8 weeks	2	−0.224(−0.525, 0.077)	0.145	71.6%	0.030

#### 3.3.7 Subgroup analyses

The subgroup analyses based on the wavelength, power, and type of laser were conducted to explore the improvement effects of different lasers on patients’ VAS pain scores, WOMAC pain scores, WOMAC function scores, and WOMAC stiffness scores in patients.

##### 3.3.7.1 Subgroup analysis according to different laser power

**Figure 10:**
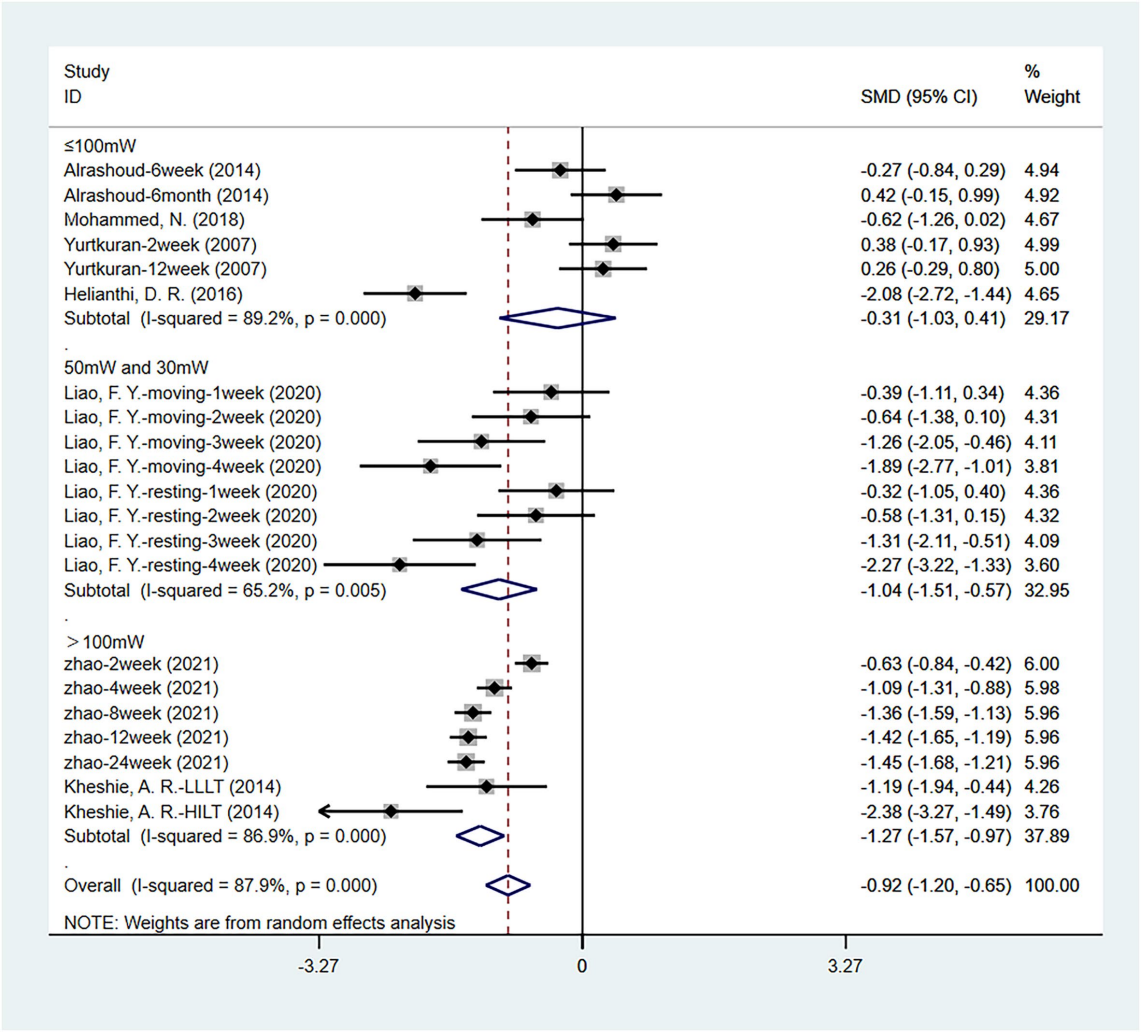
Subgroup analysis of VAS scores of different laser power.

A total of seven articles (Al Rashoud et al., 2014; Helianthi et al., 2016; Kheshie et al., 2014; Liao et al., 2020; Mohammed et al., 2018; Yurtkuran et al., 2007; Zhao et al., 2021) analyzed the VAS pain scores of the laser acupuncture and control group at different laser power. Four studies (Al Rashoud et al., 2014; Helianthi et al., 2016; Mohammed et al., 2018; Yurtkuran et al., 2007) used laser devices with a power of less than 100 mW, two studies (Kheshie et al., 2014; Zhao et al., 2021) used laser devices with a power of more than 100 mW, and one study (Liao et al., 2020) combined the use of 50 and 30 mW laser devices. The results (Figure 10) showed no statistically significant difference between the two groups when the power was less than 100 mW [SMD = −0.308, 95% CI (−1.031, 0.415), *p* = 0.403]. The use of laser devices with power greater than 100 mW (*p* = 0.000) or the combination of 50 mW and 30 mW laser devices (*p* = 0.000) may be superior to laser devices with power less than 100 mW (*p* = 0.403) in VAS improvement. There was statistically significant heterogeneity between the studies, so sensitivity analysis and publication bias measures were taken to diminish these effects.

Similar results were obtained when WOMAC pain scores, WOMAC function scores, and WOMAC stiffness scores were used for evaluation. As shown in Table 3, more effective treatment outcomes will be obtained when the laser power is greater than 100 mW.

**Table 3:** Subgroup analysis of different laser power.

Outcome	Subgroup (power)	No. of trials	Standard mean difference (95% CI)	*P*-value	I2(%)	I2 *P*-value
WOMAC pain scores	36 mW and 200 mW	2	−0.312(−0.678, 0.055)	0.095	0.0%	0.732
	≤100 mW	2	0.122(−0.078, 0.322)	0.231	0.0%	0.441
	>100 mW	2	−0.738(−0.980, −0.497)	0.000	81.9%	0.000
WOMAC function scores	36 mW and 200 mW	2	−0.192(−0.558, 0.174)	0.303	0.0%	0.471
	≤100 mW	2	0.336(−0.050,0.721)	0.088	69.3%	0.021
	>100 mW	2	−0.653(−0.930, −0.376)	0.000	86.4%	0.000
WOMAC stiffness scores	36 mW and 200 mW	2	−0.296(−0.662, 0.071)	0.114	0.0%	0.677
	≤100 mW	1	0.479(0.089, 0.870)	0.016	0.0%	0.666
	>100 mW	2	−0.314(0.434, −0.194)	0.000	35.7%	0.156

##### 3.3.7.2 Subgroup analysis according to different laser wavelengths

We included a total of nine studies, divided into different subgroups based on the wavelength of the laser used. As shown in Table 4, patients in the laser wavelength > 1,000 nm group had better improvements in VAS scores, WOMAC pain scores, WOMAC function scores, and WOMAC stiffness scores.

**Table 4:** Subgroup analysis of different laser wavelengths.

Outcome	Subgroup (wavelength)	No. of trials	Standard mean difference (95% CI)	*P*-value	I2(%)	I2 *P*-value
VAS	780 nm and 830 nm	1	−1.037(−1.508, −0.566)	0.000	65.2%	0.005
	≤1,000 nm	5	−0.427(−1.096, 0.242)	0.211	88.5%	0.000
	>1,000 nm	2	−1.281(−1.599, −0.963)	0.000	89.1%	0.000
WOMAC pain scores	650 ~ 660 nm and 10,600 nm	2	−0.312(−0.678, 0.055)	0.095	0.0%	0.732
	≤1,000 nm	2	−0.119(−1.069, 0.830)	0.805	86.2%	0.001
	>1,000 nm	2	−0.698(−0.944, −0.453)	0.000	83.5%	0.000
WOMAC function scores	650 ~ 660 nm and 10,600 nm	2	−0.192(−0.558, 0.174)	0.303	0.0%	0.471
	≤1,000 nm	2	−0.005(−1.359, 1.350)	0.995	92.7%	0.000
	>1,000 nm	2	−0.561(−0.827, −0.295)	0.000	86.2%	0.000
WOMAC stiffness scores	650 ~ 660 nm and 10,600 nm	2	−0.296(−0.662, 0.071)	0.114	0.0%	0.677
	≤1,000 nm	1	0.275(−0.178, 0.728)	0.234	42.8%	0.174
	>1,000 nm	2	−0.318(−0.448, −0.188)	0.000	46.3%	0.098

##### 3.3.7.3 Subgroup analysis according to different types of laser devices

The types of lasers used in the included studies include semiconductor lasers, CO2 lasers, solid-state lasers, and the combination of semiconductor lasers and CO2 lasers. As shown in Table 5. Among the CO2 laser and solid-state laser subgroups, there were statistically significant differences in VAS scores, WOMAC pain scores, WOMAC function scores, and WOMAC stiffness scores. The high-power solid-state laser may be able to provide better treatment outcomes. However, only one paper has analyzed it, and more studies and larger sample sizes are needed to prove it.

**Table 5:** Subgroup analysis of different laser devices.

Outcome	Subgroup (laser device)	No. of trials	Standard mean difference (95% CI)	*P*-value	I2(%)	I2 *P*-value
VAS	Semiconductor laser	6	−0.736(−1.182, −0.311)	0.001	83.5%	0.000
	CO2 laser	1	−1.188(−1.498, −0.877)	0.000	89.7%	0.000
	Solid-state laser	1	−2.381(−3.275, −1.487)	0.000	.%	.
WOMAC pain scores	Semiconductor laser and CO2 laser	2	−0.312(−0.678, 0.055)	0.095	0.0%	0.732
	Semiconductor laser	2	−0.018(−0.415, 0.379)	0.929	72.5%	0.006
	CO2 laser	2	−0.591(−0.758, −0.425)	0.000	68.5%	0.013
	Solid-state laser	1	−2 0.592(−3.521, −1.663)	0.000	.%	.
WOMAC function scores	Semiconductor laser and CO2 laser	2	−0.192(−0.558, 0.174)	0.303	0.0%	0.471
	Semiconductor laser	2	0.051(−0.511, 0.613)	0.858	86.0%	0.000
	CO2 laser	2	−0.422(−0.570, −0.275)	0.000	60.6%	0.038
	Solid-state laser	1	−3.080(−4.097, −2.063)	0.000	.%	.
WOMAC stiffness scores	Semiconductor laser and CO2 laser	2	−0.296(−0.662, 0.071)	0.114	0.0%	0.677
	Semiconductor laser	1	0.275(−0.178, 0.728)	0.234	42.8%	0.174
	CO2 laser	2	−0.292(−0.384, −0.200)	0.000	0.0%	0.455
	Solid-state laser	1	−1.186(−1.917, −0.454)	0.001	.%	.

### 3.4 Adverse event

Only one study (Shen et al., 2009) reported the adverse event. Patients had an area of reddened skin 2 mm in diameter at the irradiated site after treatment. The redness disappeared within a few hours, without treatment. It could be a normal reaction to laser exposure. No other adverse reactions were observed. In other studies of laser acupuncture, only a few patients reported mild discomfort (Choi et al., 2018; Wu et al., 2024; Rajai Firouzabadi et al., 2024). In Johannsen et al. (1994) study, one patient in the treatment group reported a burning sensation at the treatment site. The possible adverse events of laser acupuncture include local redness and swelling, skin irritation, pain aggravation, etc. Future studies are needed to further demonstrate the safety of laser acupuncture.

### 3.5 Published bias

Publication bias was assessed by an Egger’s test for change in VAS pain scores, change in WOMAC pain scores, change in WOMAC function scores, and change in WOMAC stiffness scores. Which showed no significant publication bias for change in VAS pain scores (*p* = 0.411), change in WOMAC pain scores (*p* = 0.641), change in WOMAC function scores (*p* = 0.870), and change in WOMAC stiffness scores (*p* = 0.597) (Supplementary Figures S5–S8).

## 4 Discussion

In this paper, we included the latest and most relevant clinical studies on laser acupuncture in the treatment of osteoarthritis with the aim of providing the latest clinical evidence. For the first time, we analyzed whether the power, wavelength, and type of laser have an impact on the treatment effectiveness of laser acupuncture. Chen et al. (2019) have previously conducted related research. They studied the effectiveness of laser acupuncture in patients with knee osteoarthritis and found that laser acupuncture can be effective in reducing knee pain in patients with knee osteoarthritis. However, it has no impact on knee function, stiffness, and patient’s quality of life. Different from the results of Chen et al., we found that there were significant differences in VAS pain scores, WOMAC pain scores, and WOMAC function scores between laser acupuncture and sham laser acupuncture groups during short-term follow-up outcomes (< 8 weeks), and the differences disappeared during long-term follow-up outcomes (≥ 8 weeks). No significant difference was observed in the degree of knee stiffness between the two groups at short-term and long-term follow-up. Laser acupuncture was able to relieve knee pain and improve function in the short-term outcomes (< 8 weeks), and this effect disappeared at long-term follow-up outcomes (≥ 8 weeks). Lasers with power greater than 100 mW and wavelengths greater than 1,000 nm can provide more significant therapeutic effects. CO2 lasers and solid-state lasers showed more significant therapeutic effects in our results than other types of lasers.

Recently, laser therapy has been widely used to treat musculoskeletal pain, including pain in pediatric kidney biopsies, lower back pain, and pain caused by knee OA (Oates et al., 2017; Yang et al., 2023). Huang et al. (2022) found that low-level laser acupuncture reduces postoperative pain and morphine consumption in older patients with total knee arthroplasty in a randomized controlled study. Traditional Chinese medicine believes that direct stimulation of acupoints can play a role in dredging meridians, dispersing blood stasis, and relieving pain (Wen et al., 2021). When the laser irradiates the acupuncture point, the local temperature increases promotes the dilation of blood vessels, and improves blood circulation (Yeh et al., 2004). Local metabolism is accelerated, promoting the resolution of inflammation and the transport of nutrients (Li, 2015). Studies by Mohammed et al. (2018) demonstrated that laser acupuncture can increase serum levels of *β*-endorphins and reduce substance P, thereby improving arthritis pain. β-endorphins are secreted by the hypothalamus and pituitary gland to exert their analgesic effects by binding to opioid receptors and inhibiting the release of gamma-aminobutyric acid (GABA) (Park et al., 2007). Falaki et al. (2014) demonstrated that laser irradiation can reduce the pain of trigeminal neuralgia by raising the pain threshold of nerve fibers.

Our study first evaluates the effects of laser power, wavelength, and type on treatment outcomes as a meta-analysis, providing evidence for exploring more effective parameters of laser acupuncture for the treatment of osteoarthritis. Secondly, this study included only randomized controlled trials, ensuring the high quality of the results of the related studies.

This study also has several limitations. Firstly, the number of studies and patients included was limited, and most of the studies only had short follow-up periods. Therefore, future studies should emphasize the selection of a larger sample size. Additionally, the long-term therapeutic effects of laser acupuncture should be investigated. Secondly, the characteristics of the enrolled studies were inherently clinically heterogeneous. Due to limited data, it is not possible to conduct a more comprehensive subgroup analysis based on different treatment options. Thirdly, we tried to study the influence of acupuncture point selection on the therapeutic effect to provide a more consistent acupuncture point scheme, but the point selections are often individualized based on the principles of individuation and dialectical therapy (Jiang et al., 2012). Finally, although high-intensity laser has shown the ability to significantly improve pain in patients with osteoarthritis, only one paper has reported the effect of high-intensity laser on osteoarthritis, and more evidence was needed.

## 5 Conclusion

The results of this study suggest that the application of laser acupuncture can improve knee pain and function in patients with osteoarthritis in the short term. The use of lasers with power greater than 100 mW and wavelength greater than 1,000 nm will result in better clinical improvement. CO2 lasers and solid-state lasers may be more effective than other types of lasers. These results and the role of solid-state lasers need to be validated in clinical studies with larger sample sizes and longer-term follow-ups.

## Data Availability

The raw data supporting the conclusions of this article will be made available by the authors, without undue reservation.
