# Supplementary material for: Efficacy and safety of laser acupuncture on osteoarthritis: a systematic review and meta-analysis
Source: Front Aging Neurosci. 2025 Jan 8;16:1462411. doi: 10.3389/fnagi.2024.1462411 (PMC11751068; doi:10.3389/fnagi.2024.1462411)
Supplement: Supplementary file 1 [file Table_1.docx]

Supplementary Material 1: Search strategy

# PubMed

| Search number | Query | Sort By | Filters | Search Details | Results |
| --- | --- | --- | --- | --- | --- |
| 7 | (((((((((((Osteoarthritis[Title/Abstract]) OR (Osteoarthritides[Title/Abstract])) OR (Osteoarthrosis[Title/Abstract])) OR (Osteoarthroses[Title/Abstract])) OR (Arthritis, Degenerative[Title/Abstract])) OR (Arthritides, Degenerative[Title/Abstract])) OR (Degenerative Arthritides[Title/Abstract])) OR (Degenerative Arthritis[Title/Abstract])) OR (Arthrosis[Title/Abstract])) OR (Arthroses[Title/Abstract])) OR (Osteoarthrosis Deformans[Title/Abstract])) AND (((((((((((((((((((Laser Therapy[Title/Abstract]) OR (Laser Therapies[Title/Abstract])) OR (Therapies, Laser[Title/Abstract])) OR (Therapy, Laser[Title/Abstract])) OR (Vaporization, Laser[Title/Abstract])) OR (Laser Vaporization[Title/Abstract])) OR (Laser Ablation[Title/Abstract])) OR (Ablation, Laser[Title/Abstract])) OR (Laser Tissue Ablation[Title/Abstract])) OR (Ablation, Laser Tissue[Title/Abstract])) OR (Tissue Ablation, Laser[Title/Abstract])) OR (Pulsed Laser Tissue Ablation[Title/Abstract])) OR (Laser Photoablation of Tissue[Title/Abstract])) OR (Nonablative Laser Treatment[Title/Abstract])) OR (Laser Treatment, Nonablative[Title/Abstract])) OR (Laser Treatments, Nonablative[Title/Abstract])) OR (Nonablative Laser Treatments[Title/Abstract])) AND ((((((((((((Acupuncture Therapy[Title/Abstract]) ) OR (Acupuncture Treatment[Title/Abstract])) OR (Acupuncture Treatments[Title/Abstract])) OR (Treatment, Acupuncture[Title/Abstract])) OR (Therapy, Acupuncture[Title/Abstract])) OR (Pharmacoacupuncture Treatment[Title/Abstract])) OR (Treatment, Pharmacoacupuncture[Title/Abstract])) OR (Pharmacoacupuncture Therapy[Title/Abstract])) OR (Therapy, Pharmacoacupuncture[Title/Abstract])) OR (Acupotomy[Title/Abstract])) OR (Acupotomies[Title/Abstract]))) OR ((laser moxibustion[Title/Abstract]) OR (laser acupuncture[Title/Abstract]))) | | | ("Osteoarthritis"[Title/Abstract] OR "Osteoarthritides"[Title/Abstract] OR "Osteoarthrosis"[Title/Abstract] OR "Osteoarthroses"[Title/Abstract] OR "arthritis degenerative"[Title/Abstract] OR (("Arthritis"[MeSH Terms] OR "Arthritis"[All Fields] OR "Arthritides"[All Fields] OR "polyarthritides"[All Fields]) AND "Degenerative"[Title/Abstract]) OR "degenerative arthritides"[Title/Abstract] OR "degenerative arthritis"[Title/Abstract] OR "Arthrosis"[Title/Abstract] OR "Arthroses"[Title/Abstract] OR "osteoarthrosis deformans"[Title/Abstract]) AND ((("laser therapy"[Title/Abstract] OR "laser therapies"[Title/Abstract] OR "therapies laser"[Title/Abstract] OR "therapy laser"[Title/Abstract] OR "vaporization laser"[Title/Abstract] OR "laser vaporization"[Title/Abstract] OR "laser ablation"[Title/Abstract] OR "ablation laser"[Title/Abstract] OR "laser tissue ablation"[Title/Abstract] OR (("ablate"[All Fields] OR "ablated"[All Fields] OR "ablates"[All Fields] OR "ablating"[All Fields] OR "Ablation"[All Fields] OR "ablational"[All Fields] OR "ablations"[All Fields]) AND "laser tissue"[Title/Abstract]) OR "tissue ablation laser"[Title/Abstract] OR "pulsed laser tissue ablation"[Title/Abstract] OR "laser photoablation of tissue"[Title/Abstract] OR "nonablative laser treatment"[Title/Abstract] OR "laser treatment nonablative"[Title/Abstract] OR ((("laser s"[All Fields] OR "lasers"[MeSH Terms] OR "lasers"[All Fields] OR "Laser"[All Fields] OR "lasered"[All Fields] OR "lasering"[All Fields]) AND ("therapeutics"[MeSH Terms] OR "therapeutics"[All Fields] OR "Treatments"[All Fields] OR "Therapy"[MeSH Subheading] OR "Therapy"[All Fields] OR "Treatment"[All Fields] OR "treatment s"[All Fields])) AND "Nonablative"[Title/Abstract]) OR "nonablative laser treatments"[Title/Abstract]) AND ("acupuncture therapy"[Title/Abstract] OR "acupuncture treatment"[Title/Abstract] OR "acupuncture treatments"[Title/Abstract] OR "treatment acupuncture"[Title/Abstract] OR "therapy acupuncture"[Title/Abstract] OR "pharmacoacupuncture treatment"[Title/Abstract] OR (("therapeutics"[MeSH Terms] OR "therapeutics"[All Fields] OR "Treatments"[All Fields] OR "Therapy"[MeSH Subheading] OR "Therapy"[All Fields] OR "Treatment"[All Fields] OR "treatment s"[All Fields]) AND "Pharmacoacupuncture"[Title/Abstract]) OR "pharmacoacupuncture therapy"[Title/Abstract] OR (("therapeutics"[MeSH Terms] OR "therapeutics"[All Fields] OR "Therapies"[All Fields] OR "Therapy"[MeSH Subheading] OR "Therapy"[All Fields] OR "therapy s"[All Fields] OR "therapys"[All Fields]) AND "Pharmacoacupuncture"[Title/Abstract]) OR "Acupotomy"[Title/Abstract] OR "Acupotomies"[Title/Abstract])) OR ("laser moxibustion"[Title/Abstract] OR "laser acupuncture"[Title/Abstract])) | 29 |
| 6 | ((((((((((Osteoarthritis[Title/Abstract]) OR (Osteoarthritides[Title/Abstract])) OR (Osteoarthrosis[Title/Abstract])) OR (Osteoarthroses[Title/Abstract])) OR (Arthritis, Degenerative[Title/Abstract])) OR (Arthritides, Degenerative[Title/Abstract])) OR (Degenerative Arthritides[Title/Abstract])) OR (Degenerative Arthritis[Title/Abstract])) OR (Arthrosis[Title/Abstract])) OR (Arthroses[Title/Abstract])) OR (Osteoarthrosis Deformans[Title/Abstract]) | | | "Osteoarthritis"[Title/Abstract] OR "Osteoarthritides"[Title/Abstract] OR "Osteoarthrosis"[Title/Abstract] OR "Osteoarthroses"[Title/Abstract] OR "arthritis degenerative"[Title/Abstract] OR (("Arthritis"[MeSH Terms] OR "Arthritis"[All Fields] OR "Arthritides"[All Fields] OR "polyarthritides"[All Fields]) AND "Degenerative"[Title/Abstract]) OR "degenerative arthritides"[Title/Abstract] OR "degenerative arthritis"[Title/Abstract] OR "Arthrosis"[Title/Abstract] OR "Arthroses"[Title/Abstract] OR "osteoarthrosis deformans"[Title/Abstract] | 102,423 |
| 5 | ((((((((((((((((((Laser Therapy[Title/Abstract]) OR (Laser Therapies[Title/Abstract])) OR (Therapies, Laser[Title/Abstract])) OR (Therapy, Laser[Title/Abstract])) OR (Vaporization, Laser[Title/Abstract])) OR (Laser Vaporization[Title/Abstract])) OR (Laser Ablation[Title/Abstract])) OR (Ablation, Laser[Title/Abstract])) OR (Laser Tissue Ablation[Title/Abstract])) OR (Ablation, Laser Tissue[Title/Abstract])) OR (Tissue Ablation, Laser[Title/Abstract])) OR (Pulsed Laser Tissue Ablation[Title/Abstract])) OR (Laser Photoablation of Tissue[Title/Abstract])) OR (Nonablative Laser Treatment[Title/Abstract])) OR (Laser Treatment, Nonablative[Title/Abstract])) OR (Laser Treatments, Nonablative[Title/Abstract])) OR (Nonablative Laser Treatments[Title/Abstract])) AND ((((((((((((Acupuncture Therapy[Title/Abstract]) ) OR (Acupuncture Treatment[Title/Abstract])) OR (Acupuncture Treatments[Title/Abstract])) OR (Treatment, Acupuncture[Title/Abstract])) OR (Therapy, Acupuncture[Title/Abstract])) OR (Pharmacoacupuncture Treatment[Title/Abstract])) OR (Treatment, Pharmacoacupuncture[Title/Abstract])) OR (Pharmacoacupuncture Therapy[Title/Abstract])) OR (Therapy, Pharmacoacupuncture[Title/Abstract])) OR (Acupotomy[Title/Abstract])) OR (Acupotomies[Title/Abstract]))) OR ((laser moxibustion[Title/Abstract]) OR (laser acupuncture[Title/Abstract])) | | | (("laser therapy"[Title/Abstract] OR "laser therapies"[Title/Abstract] OR "therapies laser"[Title/Abstract] OR "therapy laser"[Title/Abstract] OR "vaporization laser"[Title/Abstract] OR "laser vaporization"[Title/Abstract] OR "laser ablation"[Title/Abstract] OR "ablation laser"[Title/Abstract] OR "laser tissue ablation"[Title/Abstract] OR (("ablate"[All Fields] OR "ablated"[All Fields] OR "ablates"[All Fields] OR "ablating"[All Fields] OR "Ablation"[All Fields] OR "ablational"[All Fields] OR "ablations"[All Fields]) AND "laser tissue"[Title/Abstract]) OR "tissue ablation laser"[Title/Abstract] OR "pulsed laser tissue ablation"[Title/Abstract] OR "laser photoablation of tissue"[Title/Abstract] OR "nonablative laser treatment"[Title/Abstract] OR "laser treatment nonablative"[Title/Abstract] OR ((("laser s"[All Fields] OR "lasers"[MeSH Terms] OR "lasers"[All Fields] OR "Laser"[All Fields] OR "lasered"[All Fields] OR "lasering"[All Fields]) AND ("therapeutics"[MeSH Terms] OR "therapeutics"[All Fields] OR "Treatments"[All Fields] OR "Therapy"[MeSH Subheading] OR "Therapy"[All Fields] OR "Treatment"[All Fields] OR "treatment s"[All Fields])) AND "Nonablative"[Title/Abstract]) OR "nonablative laser treatments"[Title/Abstract]) AND ("acupuncture therapy"[Title/Abstract] OR "acupuncture treatment"[Title/Abstract] OR "acupuncture treatments"[Title/Abstract] OR "treatment acupuncture"[Title/Abstract] OR "therapy acupuncture"[Title/Abstract] OR "pharmacoacupuncture treatment"[Title/Abstract] OR (("therapeutics"[MeSH Terms] OR "therapeutics"[All Fields] OR "Treatments"[All Fields] OR "Therapy"[MeSH Subheading] OR "Therapy"[All Fields] OR "Treatment"[All Fields] OR "treatment s"[All Fields]) AND "Pharmacoacupuncture"[Title/Abstract]) OR "pharmacoacupuncture therapy"[Title/Abstract] OR (("therapeutics"[MeSH Terms] OR "therapeutics"[All Fields] OR "Therapies"[All Fields] OR "Therapy"[MeSH Subheading] OR "Therapy"[All Fields] OR "therapy s"[All Fields] OR "therapys"[All Fields]) AND "Pharmacoacupuncture"[Title/Abstract]) OR "Acupotomy"[Title/Abstract] OR "Acupotomies"[Title/Abstract])) OR ("laser moxibustion"[Title/Abstract] OR "laser acupuncture"[Title/Abstract]) | 436 |
| 4 | (laser moxibustion[Title/Abstract]) OR (laser acupuncture[Title/Abstract]) | | | "laser moxibustion"[Title/Abstract] OR "laser acupuncture"[Title/Abstract] | 419 |
| 3 | (((((((((((((((((Laser Therapy[Title/Abstract]) OR (Laser Therapies[Title/Abstract])) OR (Therapies, Laser[Title/Abstract])) OR (Therapy, Laser[Title/Abstract])) OR (Vaporization, Laser[Title/Abstract])) OR (Laser Vaporization[Title/Abstract])) OR (Laser Ablation[Title/Abstract])) OR (Ablation, Laser[Title/Abstract])) OR (Laser Tissue Ablation[Title/Abstract])) OR (Ablation, Laser Tissue[Title/Abstract])) OR (Tissue Ablation, Laser[Title/Abstract])) OR (Pulsed Laser Tissue Ablation[Title/Abstract])) OR (Laser Photoablation of Tissue[Title/Abstract])) OR (Nonablative Laser Treatment[Title/Abstract])) OR (Laser Treatment, Nonablative[Title/Abstract])) OR (Laser Treatments, Nonablative[Title/Abstract])) OR (Nonablative Laser Treatments[Title/Abstract])) AND ((((((((((((Acupuncture Therapy[Title/Abstract]) ) OR (Acupuncture Treatment[Title/Abstract])) OR (Acupuncture Treatments[Title/Abstract])) OR (Treatment, Acupuncture[Title/Abstract])) OR (Therapy, Acupuncture[Title/Abstract])) OR (Pharmacoacupuncture Treatment[Title/Abstract])) OR (Treatment, Pharmacoacupuncture[Title/Abstract])) OR (Pharmacoacupuncture Therapy[Title/Abstract])) OR (Therapy, Pharmacoacupuncture[Title/Abstract])) OR (Acupotomy[Title/Abstract])) OR (Acupotomies[Title/Abstract])) | | | ("laser therapy"[Title/Abstract] OR "laser therapies"[Title/Abstract] OR "therapies laser"[Title/Abstract] OR "therapy laser"[Title/Abstract] OR "vaporization laser"[Title/Abstract] OR "laser vaporization"[Title/Abstract] OR "laser ablation"[Title/Abstract] OR "ablation laser"[Title/Abstract] OR "laser tissue ablation"[Title/Abstract] OR (("ablate"[All Fields] OR "ablated"[All Fields] OR "ablates"[All Fields] OR "ablating"[All Fields] OR "Ablation"[All Fields] OR "ablational"[All Fields] OR "ablations"[All Fields]) AND "laser tissue"[Title/Abstract]) OR "tissue ablation laser"[Title/Abstract] OR "pulsed laser tissue ablation"[Title/Abstract] OR "laser photoablation of tissue"[Title/Abstract] OR "nonablative laser treatment"[Title/Abstract] OR "laser treatment nonablative"[Title/Abstract] OR ((("laser s"[All Fields] OR "lasers"[MeSH Terms] OR "lasers"[All Fields] OR "Laser"[All Fields] OR "lasered"[All Fields] OR "lasering"[All Fields]) AND ("therapeutics"[MeSH Terms] OR "therapeutics"[All Fields] OR "Treatments"[All Fields] OR "Therapy"[MeSH Subheading] OR "Therapy"[All Fields] OR "Treatment"[All Fields] OR "treatment s"[All Fields])) AND "Nonablative"[Title/Abstract]) OR "nonablative laser treatments"[Title/Abstract]) AND ("acupuncture therapy"[Title/Abstract] OR "acupuncture treatment"[Title/Abstract] OR "acupuncture treatments"[Title/Abstract] OR "treatment acupuncture"[Title/Abstract] OR "therapy acupuncture"[Title/Abstract] OR "pharmacoacupuncture treatment"[Title/Abstract] OR (("therapeutics"[MeSH Terms] OR "therapeutics"[All Fields] OR "Treatments"[All Fields] OR "Therapy"[MeSH Subheading] OR "Therapy"[All Fields] OR "Treatment"[All Fields] OR "treatment s"[All Fields]) AND "Pharmacoacupuncture"[Title/Abstract]) OR "pharmacoacupuncture therapy"[Title/Abstract] OR (("therapeutics"[MeSH Terms] OR "therapeutics"[All Fields] OR "Therapies"[All Fields] OR "Therapy"[MeSH Subheading] OR "Therapy"[All Fields] OR "therapy s"[All Fields] OR "therapys"[All Fields]) AND "Pharmacoacupuncture"[Title/Abstract]) OR "Acupotomy"[Title/Abstract] OR "Acupotomies"[Title/Abstract]) | 33 |
| 2 | (((((((((((Acupuncture Therapy[Title/Abstract]) ) OR (Acupuncture Treatment[Title/Abstract])) OR (Acupuncture Treatments[Title/Abstract])) OR (Treatment, Acupuncture[Title/Abstract])) OR (Therapy, Acupuncture[Title/Abstract])) OR (Pharmacoacupuncture Treatment[Title/Abstract])) OR (Treatment, Pharmacoacupuncture[Title/Abstract])) OR (Pharmacoacupuncture Therapy[Title/Abstract])) OR (Therapy, Pharmacoacupuncture[Title/Abstract])) OR (Acupotomy[Title/Abstract])) OR (Acupotomies[Title/Abstract]) | | | "acupuncture therapy"[Title/Abstract] OR "acupuncture treatment"[Title/Abstract] OR "acupuncture treatments"[Title/Abstract] OR "treatment acupuncture"[Title/Abstract] OR "therapy acupuncture"[Title/Abstract] OR "pharmacoacupuncture treatment"[Title/Abstract] OR (("therapeutics"[MeSH Terms] OR "therapeutics"[All Fields] OR "Treatments"[All Fields] OR "Therapy"[MeSH Subheading] OR "Therapy"[All Fields] OR "Treatment"[All Fields] OR "treatment s"[All Fields]) AND "Pharmacoacupuncture"[Title/Abstract]) OR "pharmacoacupuncture therapy"[Title/Abstract] OR (("therapeutics"[MeSH Terms] OR "therapeutics"[All Fields] OR "therapies"[All Fields] OR "Therapy"[MeSH Subheading] OR "Therapy"[All Fields] OR "therapy s"[All Fields] OR "therapys"[All Fields]) AND "Pharmacoacupuncture"[Title/Abstract]) OR "Acupotomy"[Title/Abstract] OR "Acupotomies"[Title/Abstract] | 5,501 |
| 1 | ((((((((((((((((Laser Therapy[Title/Abstract]) OR (Laser Therapies[Title/Abstract])) OR (Therapies, Laser[Title/Abstract])) OR (Therapy, Laser[Title/Abstract])) OR (Vaporization, Laser[Title/Abstract])) OR (Laser Vaporization[Title/Abstract])) OR (Laser Ablation[Title/Abstract])) OR (Ablation, Laser[Title/Abstract])) OR (Laser Tissue Ablation[Title/Abstract])) OR (Ablation, Laser Tissue[Title/Abstract])) OR (Tissue Ablation, Laser[Title/Abstract])) OR (Pulsed Laser Tissue Ablation[Title/Abstract])) OR (Laser Photoablation of Tissue[Title/Abstract])) OR (Nonablative Laser Treatment[Title/Abstract])) OR (Laser Treatment, Nonablative[Title/Abstract])) OR (Laser Treatments, Nonablative[Title/Abstract])) OR (Nonablative Laser Treatments[Title/Abstract]) | | | "laser therapy"[Title/Abstract] OR "laser therapies"[Title/Abstract] OR "therapies laser"[Title/Abstract] OR "therapy laser"[Title/Abstract] OR "vaporization laser"[Title/Abstract] OR "laser vaporization"[Title/Abstract] OR "laser ablation"[Title/Abstract] OR "ablation laser"[Title/Abstract] OR "laser tissue ablation"[Title/Abstract] OR (("ablate"[All Fields] OR "ablated"[All Fields] OR "ablates"[All Fields] OR "ablating"[All Fields] OR "Ablation"[All Fields] OR "ablational"[All Fields] OR "ablations"[All Fields]) AND "laser tissue"[Title/Abstract]) OR "tissue ablation laser"[Title/Abstract] OR "pulsed laser tissue ablation"[Title/Abstract] OR "laser photoablation of tissue"[Title/Abstract] OR "nonablative laser treatment"[Title/Abstract] OR "laser treatment nonablative"[Title/Abstract] OR ((("laser s"[All Fields] OR "lasers"[MeSH Terms] OR "lasers"[All Fields] OR "Laser"[All Fields] OR "lasered"[All Fields] OR "lasering"[All Fields]) AND ("therapeutics"[MeSH Terms] OR "therapeutics"[All Fields] OR "Treatments"[All Fields] OR "Therapy"[MeSH Subheading] OR "Therapy"[All Fields] OR "Treatment"[All Fields] OR "treatment s"[All Fields])) AND "Nonablative"[Title/Abstract]) OR "nonablative laser treatments"[Title/Abstract] | 23,553 |

# Embase

| No. | Query | Results |
| --- | --- | --- |
| #47 | #34 AND #46 | 39 |
| #46 | #35 OR #36 OR #37 OR #38 OR #39 OR #40 OR #41 OR #42 OR #43 OR #44 OR #45 | 135831 |
| #45 | 'osteoarthrosis deformans':ab,ti | 156 |
| #44 | 'arthroses':ab,ti | 614 |
| #43 | 'arthrosis':ab,ti | 7880 |
| #42 | 'degenerative arthritis':ab,ti | 1724 |
| #41 | 'degenerative arthritides':ab,ti | 16 |
| #40 | 'arthritides, degenerative':ab,ti | 1 |
| #39 | 'arthritis, degenerative':ab,ti | 82 |
| #38 | 'osteoarthroses':ab,ti | 38 |
| #37 | 'osteoarthrosis':ab,ti | 4519 |
| #36 | 'osteoarthritides':ab,ti | 5 |
| #35 | 'osteoarthritis':ab,ti | 123054 |
| #34 | #31 OR #32 OR #33 | 597 |
| #33 | 'laser acupuncture':ab,ti | 553 |
| #32 | 'laser moxibustion':ab,ti | 26 |
| #31 | #18 AND #30 | 38 |
| #30 | #19 OR #20 OR #21 OR #22 OR #23 OR #24 OR #25 OR #26 OR #27 OR #28 OR #29 | 7524 |
| #29 | 'acupotomies':ab,ti | 0 |
| #28 | 'acupotomy':ab,ti | 185 |
| #27 | 'therapy, pharmacoacupuncture':ab,ti | 0 |
| #26 | 'pharmacoacupuncture therapy':ab,ti | 3 |
| #25 | 'treatment, pharmacoacupuncture':ab,ti | 0 |
| #24 | 'pharmacoacupuncture treatment':ab,ti | 1 |
| #23 | 'therapy, acupuncture':ab,ti | 336 |
| #22 | 'treatment, acupuncture':ab,ti | 245 |
| #21 | 'acupuncture treatments':ab,ti | 788 |
| #20 | 'acupuncture treatment':ab,ti | 4830 |
| #19 | 'acupuncture therapy':ab,ti | 2140 |
| #18 | #1 OR #2 OR #3 OR #4 OR #5 OR #6 OR #7 OR #8 OR #9 OR #10 OR #11 OR #12 OR #13 OR #14 OR #15 OR #16 OR #17 | 26371 |
| #17 | 'nonablative laser treatments':ab,ti | 13 |
| #16 | 'laser treatments, nonablative':ab,ti | 0 |
| #15 | 'laser treatment, nonablative':ab,ti | 2 |
| #14 | 'nonablative laser treatment':ab,ti | 25 |
| #13 | 'laser photoablation of tissue':ab,ti | 0 |
| #12 | 'pulsed laser tissue ablation':ab,ti | 0 |
| #11 | 'tissue ablation, laser':ab,ti | 4 |
| #10 | 'ablation, laser tissue':ab,ti | 0 |
| #9 | 'laser tissue ablation':ab,ti | 26 |
| #8 | 'ablation, laser':ab,ti | 190 |
| #7 | 'laser vaporization':ab,ti | 1406 |
| #6 | 'laser ablation':ab,ti | 10864 |
| #5 | 'vaporization, laser':ab,ti | 20 |
| #4 | 'therapy, laser':ab,ti | 373 |
| #3 | 'therapies, laser':ab,ti | 48 |
| #2 | 'laser therapies':ab,ti | 402 |
| #1 | 'laser therapy':ab,ti | 13884 |

# Web of Science

# Searches:

1: (((((((((((((((ALL=(Laser Therapy)) OR ALL=(Laser Therapies)) OR ALL=(Therapies, Laser)) OR ALL=(Therapy, Laser)) OR ALL=(Vaporization, Laser)) OR ALL=(Laser Vaporization)) OR ALL=(Laser Ablation)) OR ALL=(Ablation, Laser)) OR ALL=(Laser Tissue Ablation)) OR ALL=(Ablation, Laser Tissue)) OR ALL=(Tissue Ablation, Laser)) OR ALL=(Pulsed Laser Tissue Ablation)) OR ALL=(Laser Photoablation of Tissue)) OR ALL=(Nonablative Laser Treatment)) OR ALL=(Laser Treatment, Nonablative)) OR ALL=(Laser Treatments, Nonablative) Date Run: Sun Dec 24 2023 12:40:50 GMT+0800 (中国标准时间) Results: 107202

2: ((((((((((ALL=(Acupuncture Therapy)) OR ALL=(Acupuncture Treatment)) OR ALL=(Acupuncture Treatments)) OR ALL=(Treatment, Acupuncture)) OR ALL=(Therapy, Acupuncture)) OR ALL=(Pharmacoacupuncture Treatment)) OR ALL=(Treatment, Pharmacoacupuncture)) OR ALL=(Pharmacoacupuncture Therapy)) OR ALL=(Therapy, Pharmacoacupuncture)) OR ALL=(Acupotomy)) OR ALL=(Acupotomies) Date Run: Sun Dec 24 2023 12:48:39 GMT+0800 (中国标准时间) Results: 17913

3: #1 AND #2 Date Run: Sun Dec 24 2023 12:51:37 GMT+0800 (中国标准时间) Results: 528

4: (ALL=(laser moxibustion)) OR ALL=(laser acupuncture ) Date Run: Sun Dec 24 2023 12:52:15 GMT+0800 (中国标准时间) Results: 976

5: #3 OR #4 Date Run: Sun Dec 24 2023 12:52:51 GMT+0800 (中国标准时间) Results: 976

6: ((((((((((ALL=(Osteoarthritis)) OR ALL=(Osteoarthritides)) OR ALL=(Osteoarthrosis)) OR ALL=(Osteoarthroses)) OR ALL=(Arthritis, Degenerative)) OR ALL=(Arthritides, Degenerative)) OR ALL=(Degenerative Arthritides)) OR ALL=(Degenerative Arthritis)) OR ALL=(Arthrosis)) OR ALL=(Arthroses)) OR ALL=(Osteoarthrosis Deformans) Date Run: Sun Dec 24 2023 12:55:38 GMT+0800 (中国标准时间) Results: 128057

7: #5 AND #6 Date Run: Sun Dec 24 2023 12:56:45 GMT+0800 (中国标准时间) Results: 75

# Cochrane library

ID Search Hits

#1 (Laser Therapy):ti,ab,kw OR (Laser Therapies):ti,ab,kw OR (Therapies, Laser):ti,ab,kw OR (Therapy, Laser):ti,ab,kw OR (Vaporization, Laser):ti,ab,kw 13214

#2 (Laser Vaporization):ti,ab,kw OR (Laser Ablation):ti,ab,kw OR (Ablation, Laser):ti,ab,kw OR (Laser Tissue Ablation):ti,ab,kw OR (Ablation, Laser Tissue):ti,ab,kw 1643

#3 (Tissue Ablation, Laser):ti,ab,kw OR (Pulsed Laser Tissue Ablation):ti,ab,kw OR (Laser Photoablation of Tissue):ti,ab,kw OR (Nonablative Laser Treatment):ti,ab,kw OR (Laser Treatment, Nonablative):ti,ab,kw 505

#4 (Laser Treatments, Nonablative):ti,ab,kw OR (Nonablative Laser Treatments):ti,ab,kw 152

#5 #1 OR #2 OR #3 OR #4 13943

#6 (Acupuncture Therapy):ti,ab,kw OR (Acupuncture Treatment):ti,ab,kw OR (Acupuncture Treatments):ti,ab,kw OR (Treatment, Acupuncture):ti,ab,kw OR (Therapy, Acupuncture):ti,ab,kw 15499

#7 (Pharmacoacupuncture Treatment):ti,ab,kw OR (Treatment, Pharmacoacupuncture):ti,ab,kw OR (Pharmacoacupuncture Therapy):ti,ab,kw OR (Therapy, Pharmacoacupuncture):ti,ab,kw OR (Acupotomy):ti,ab,kw 122

#8 Acupotomies 0

#9 #6 OR #7 OR #8 15553

#10 #5 AND #9 499

#11 laser acupuncture 885

#12 laser moxibustion 141

#13 #10 OR #11 OR #12 903

#14 (Osteoarthritis):ti,ab,kw OR (Osteoarthritides):ti,ab,kw OR (Osteoarthrosis):ti,ab,kw OR (Osteoarthroses):ti,ab,kw OR (Arthritis, Degenerative):ti,ab,kw 23769

#15 (Arthritides, Degenerative):ti,ab,kw OR (Degenerative Arthritides):ti,ab,kw OR (Degenerative Arthritis):ti,ab,kw OR (Arthrosis):ti,ab,kw OR (Arthroses):ti,ab,kw 1121

#16 Osteoarthrosis Deformans 8

#17 #14 OR #15 OR #16 24033

#18 #13 AND #17 60
