# Supplementary figures and images for: Efficacy and safety of laser acupuncture on osteoarthritis: a systematic review and meta-analysis
Source: Front Aging Neurosci. 2025 Jan 8;16:1462411. doi: 10.3389/fnagi.2024.1462411 (PMC11751068; doi:10.3389/fnagi.2024.1462411)

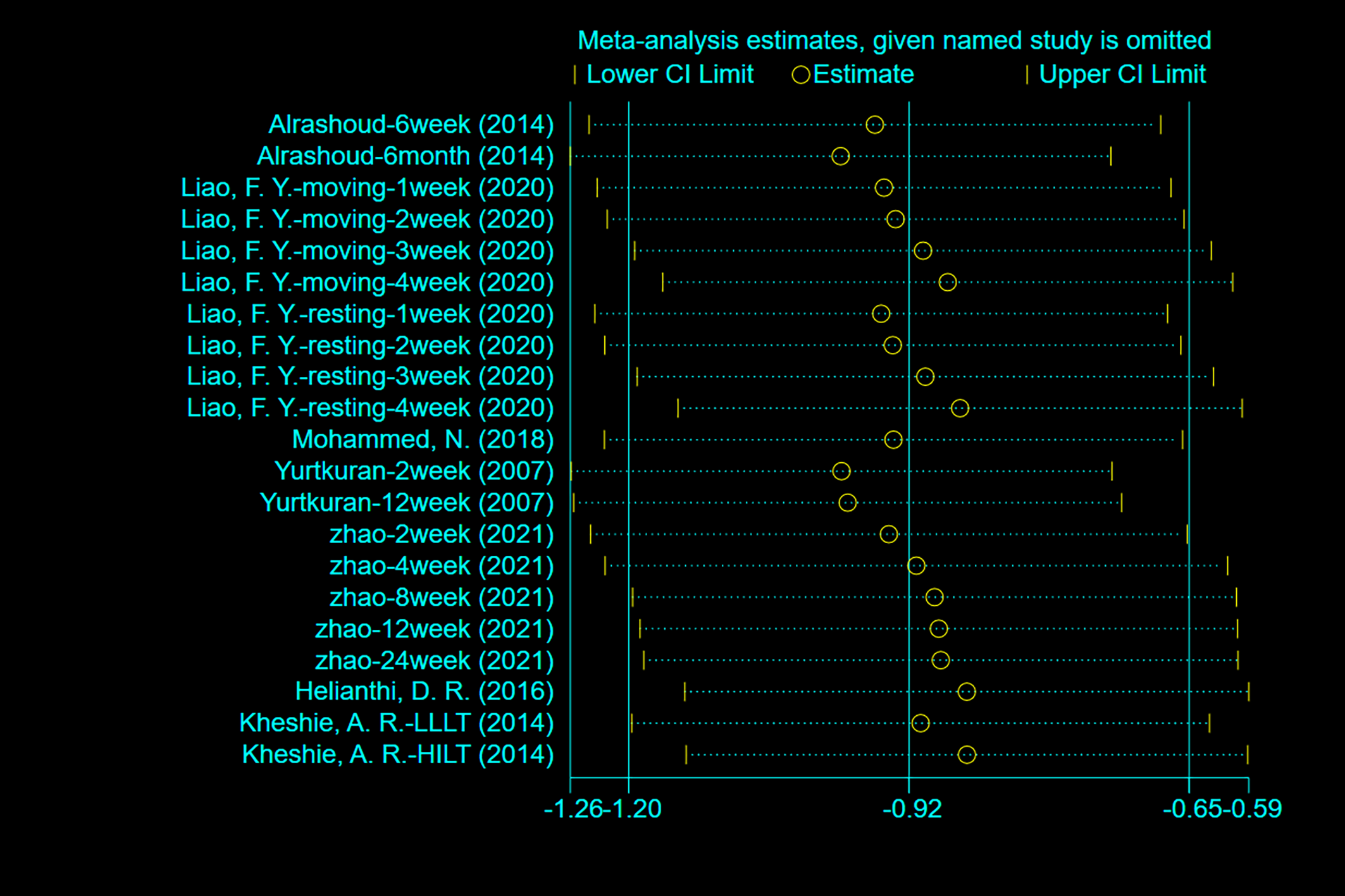

Supplement: Supplementary file 2 [file Image_1.tif]

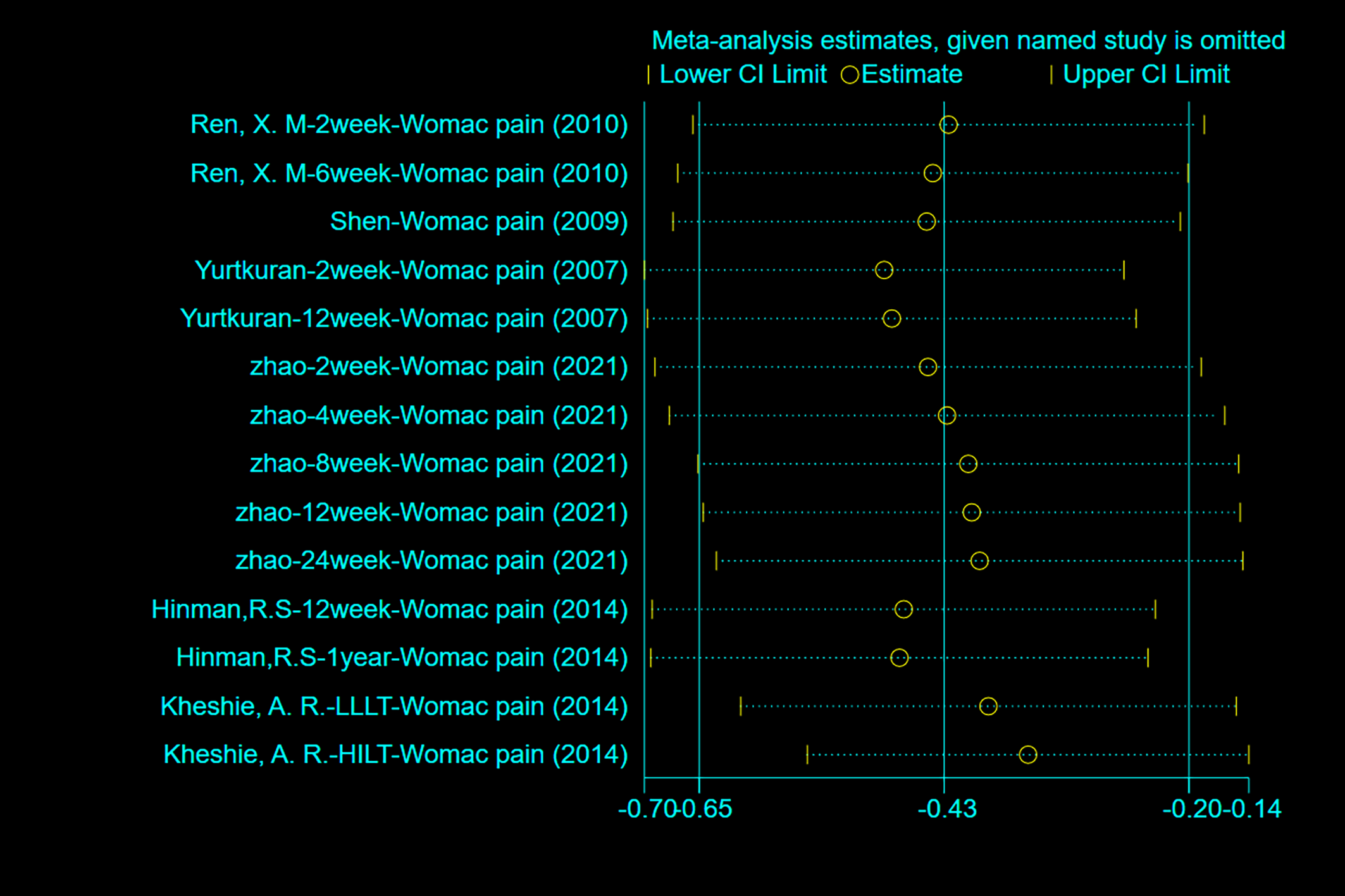

Supplement: Supplementary file 3 [file Image_2.tif]

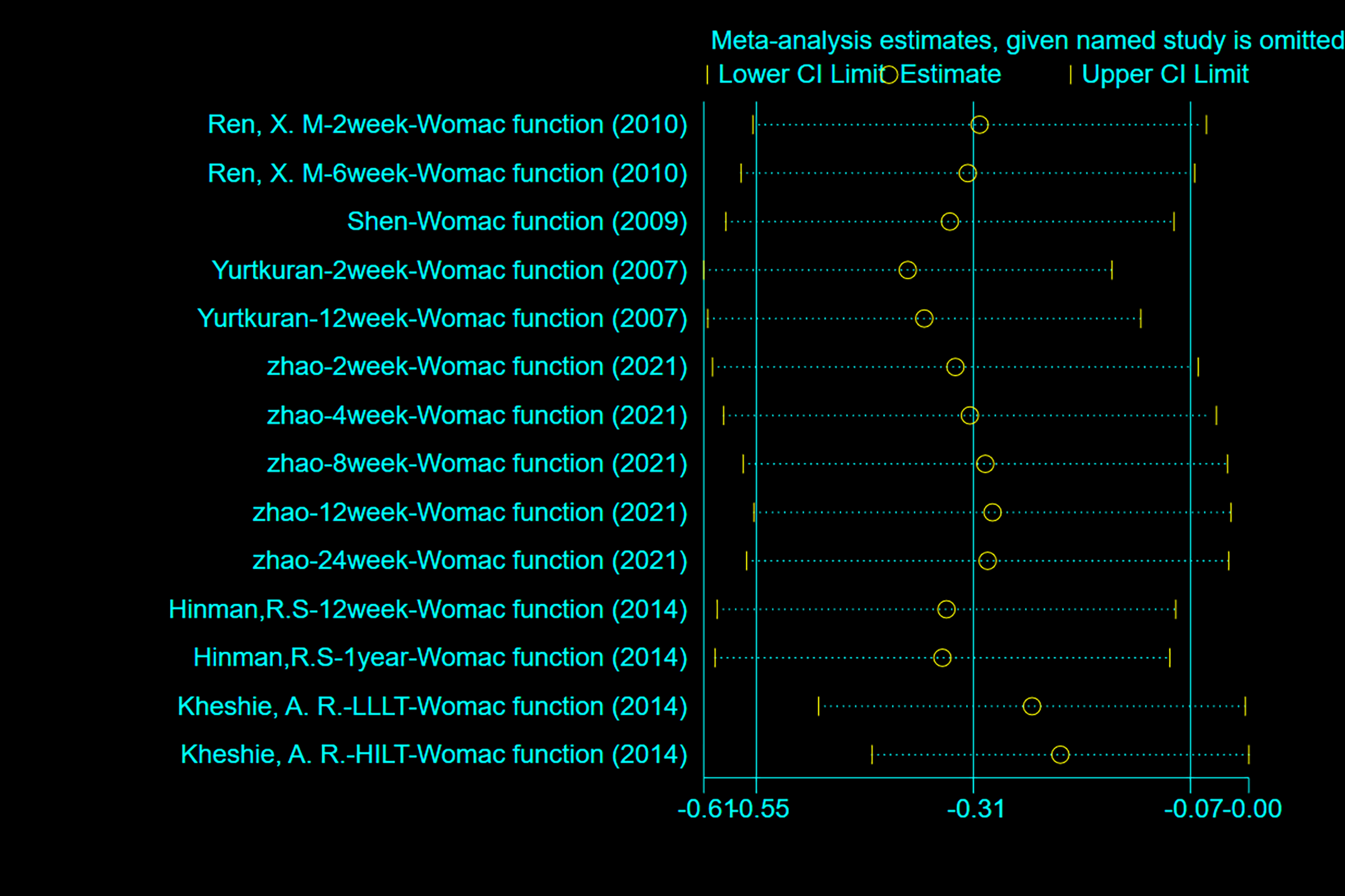

Supplement: Supplementary file 4 [file Image_3.tif]

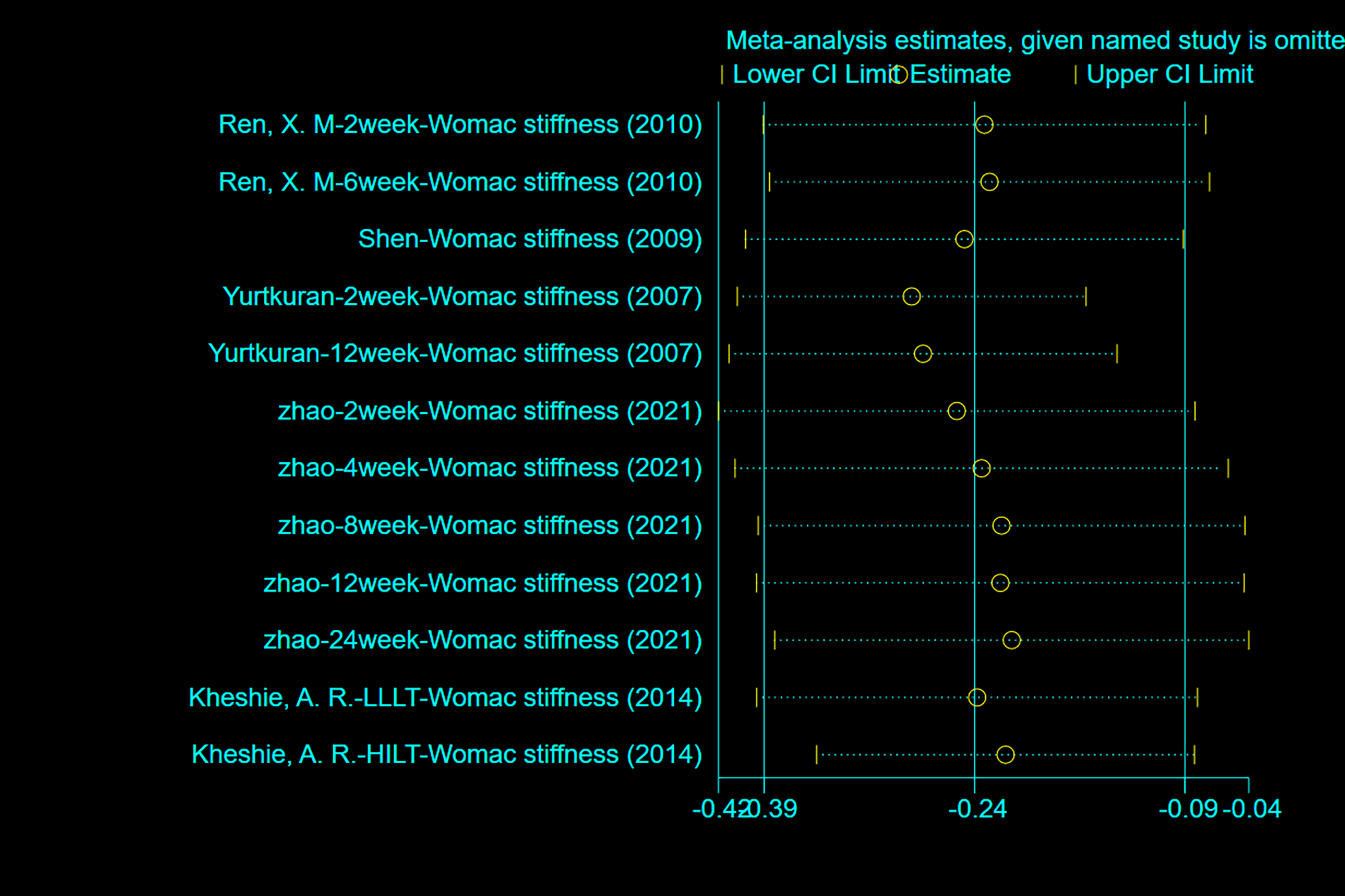

Supplement: Supplementary file 5 [file Image_4.tif]

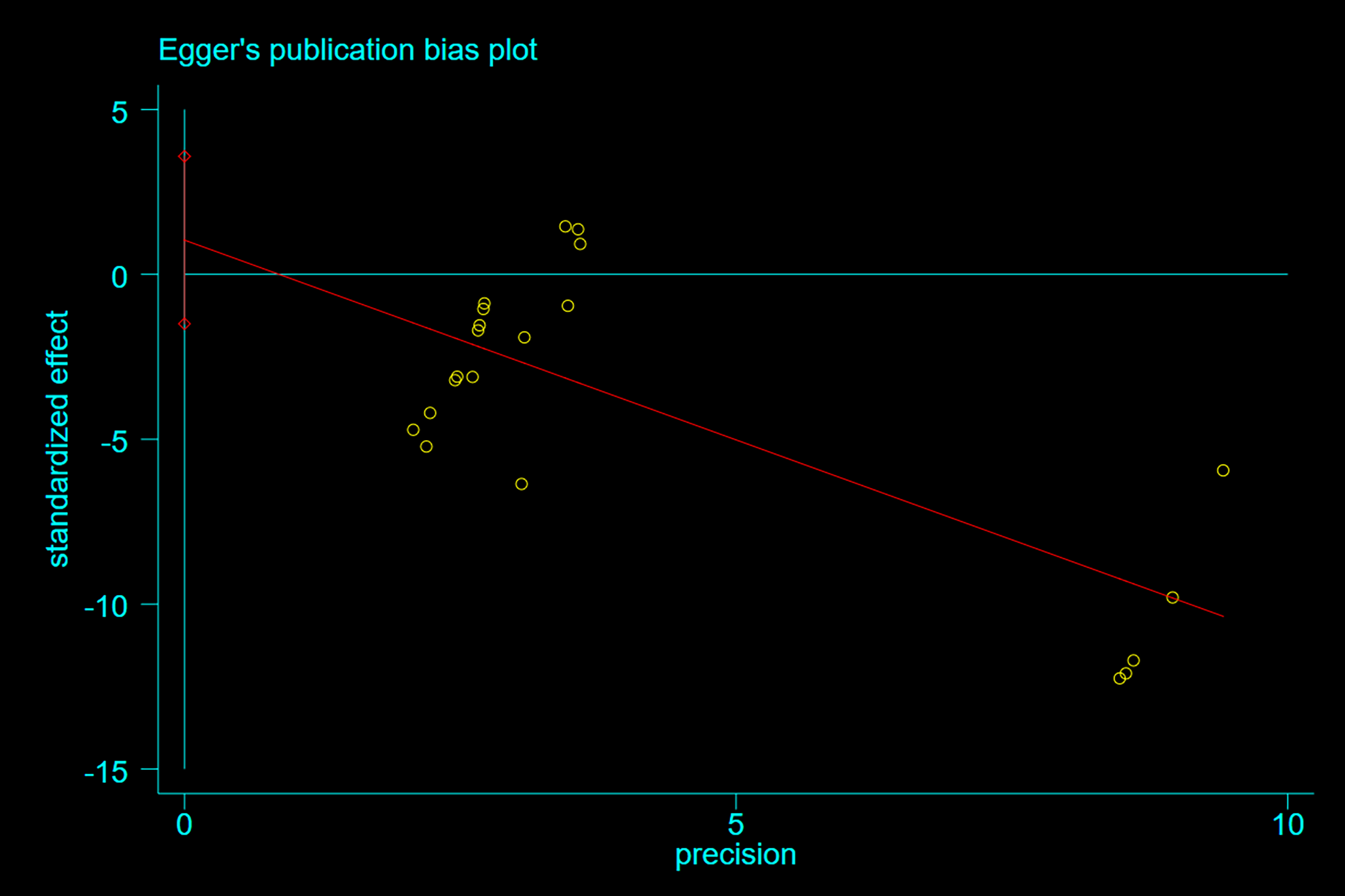

Supplement: Supplementary file 6 [file Image_5.tif]

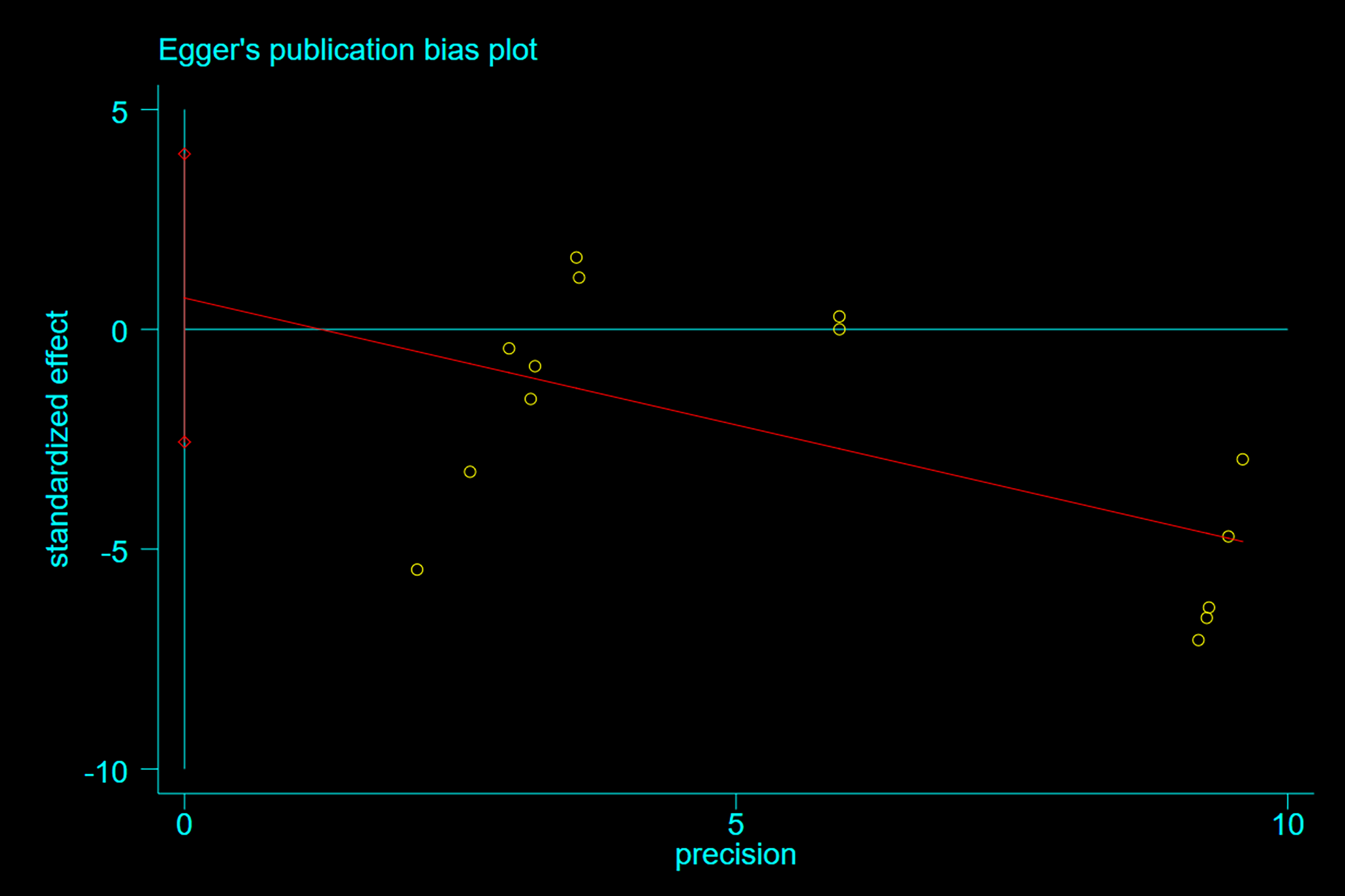

Supplement: Supplementary file 7 [file Image_6.tif]

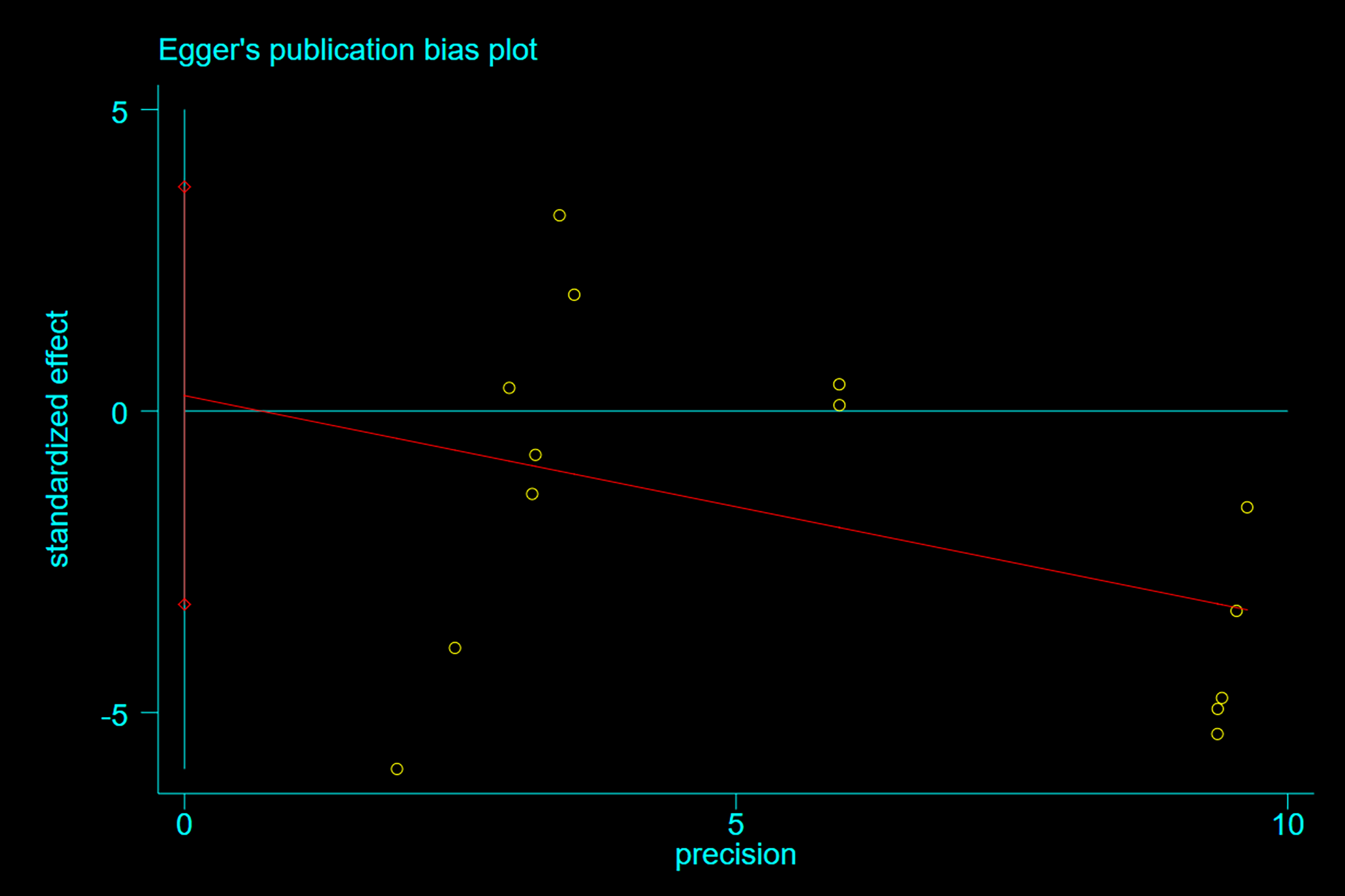

Supplement: Supplementary file 8 [file Image_7.tif]

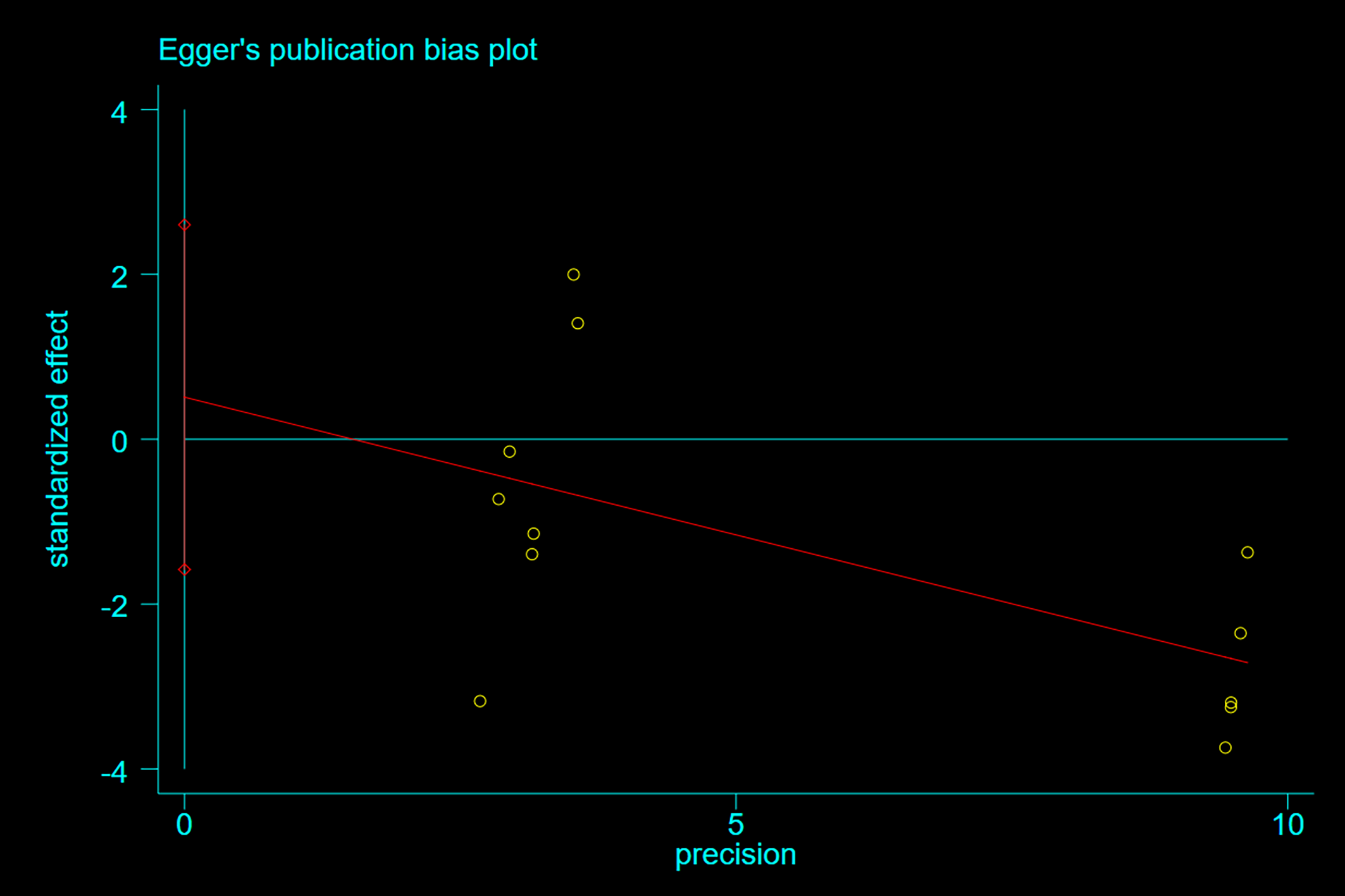

Supplement: Supplementary file 9 [file Image_8.tif]
